# *PRINSAS 2.0*: a Python-based graphical user interface tool for fitting polydisperse spherical pore models in small-angle scattering analysis of porous materials

**DOI:** 10.1107/S1600576725004315

**Published:** 2025-07-02

**Authors:** Phung Nhu Hao Vu, Andrzej P. Radlinski, Tomasz Blach, John Daniels, Klaus Regenauer-Lieb

**Affiliations:** a School of Materials Science and Engineering UNSW Sydney Sydney New South Wales2052 Australia; b WA School of Mines: Minerals, Energy and Chemical Engineering Curtin University Perth Western Australia6102 Australia; c Queensland Micro Nanotechnology Centre Griffith University Nathan Queensland4111 Australia; Australian Synchrotron, ANSTO, Australia

**Keywords:** small-angle scattering, polydisperse spherical pore models, porous media, data analysis software, *PRINSAS 2.0*

## Abstract

Despite the growing use of small- and ultra-small-angle scattering (SAS/USAS) across various fields, data processing remains challenging due to the complexity of SAS analysis and the limited accessibility of existing analysis software. These issues are addressed with *PRINSAS 2.0*, a portable Python-based tool with an intuitive graphical user interface. It enables efficient fitting of the polydisperse spherical pore model to SAS data and is specifically designed for porous materials often encountered in geoscience. This paper outlines the scientific and mathematical foundations of the software, along with its numerical implementation, to provide users with theoretical context and to support future development. The software was tested and validated using data from a range of geological and engineered porous samples measured at various neutron scattering facilities, ensuring broad compatibility. Additional validation using synthetic data sets, along with comparisons with existing pore size distribution fitting tools, confirmed its robustness in recovering predefined pore size distributions. *PRINSAS 2.0* offers wide accessibility while ensuring that the fit results adhere closely to the underlying theoretical model, making it a practical tool for non-specialist users of SAS techniques. It also integrates seamlessly with larger Python-based SAS analysis frameworks, while remaining fully functional as a standalone application.

## Abstract 2

The program *PRINSAS 2.0* is designed to fit the polydisperse spherical pore model to small-angle scattering (SAS) data of porous materials. It improves accessibility for users without extensive SAS analysis or programming experience, while being optimized for porous systems commonly found in geological samples. The fitting algorithm is validated on experimental and synthetic data sets to ensure broad applicability, and is benchmarked against other tools in the field for acquiring size distribution.

## 1. Introduction

Recent advances in neutron source technology – including gains in flux intensity (Oak Ridge National Laboratory, 2023 ▸; ANSTO, 2024 ▸) and enhanced stability through more rigorously reviewed practices (International Atomic Energy Agency, 2024 ▸) – have established small- and ultra-small-angle scattering (SAS/USAS) as powerful techniques for studying micro- and nanoscale structures. These advancements have led to (i) improved reliability, (ii) faster data acquisition enabling sub-millisecond temporal sampling, and (iii) increased resolution and signal-to-noise ratio, allowing studies from ångström to micrometre scales. The integration of automatic multi-sample changers has further improved the user experience and increased sample throughput (Rehm *et al.*, 2018 ▸; Wood *et al.*, 2018 ▸).

Originally adopted by the biophysical community for protein studies (Engelman & Moore, 1975 ▸), SAS later found widespread application in materials science (Schelten *et al.*, 1976 ▸) and geology (Radlinski *et al.*, 1996 ▸), particularly for analysing porous structures and nanoscale sorption behaviour. One of SAS’s key advantages is its non-destructive nature (Radlinski, 2006 ▸), and this coupled with the ability to distinguish between open and closed porosity through contrast matching techniques (Bahadur *et al.*, 2018 ▸; Radlinski *et al.*, 2021 ▸; Vu *et al.*, 2024 ▸) makes it especially valuable for studying complex material systems.

Despite these experimental advancements, data analysis remains a critical bottleneck. Modern SAS instruments generate tens to hundreds of gigabytes of multidimensional data sets [*e.g.I*(*Q*, *t*) for time-resolved studies], yet extracting physically meaningful parameters remains labour intensive and often requires a high level of expertise. Traditionally, a strong foundation in mathematical computing was essential for the effective interpretation of these large data sets. However, over the years, various software packages have been developed to streamline this process, which can be broadly classified two main categories:

(i) Large-scale software, ranging from (*a*) internationally developed open-source projects such as *SASView* (https://www.sasview.org/) and *Mantid* (Arnold *et al.*, 2014 ▸) to (*b*) facility-developed suites like *Irena* (Ilavsky & Jemian, 2009 ▸), *GSAS-II* (Toby & Von Dreele, 2013 ▸; Von Dreele, 2014 ▸) and *SAXSutilities2*(https://www.saxsutilities.eu/mediawiki/index.php?title=Overview). These programs offer comprehensive functionality – including both data reduction and analysis – but often come with steep learning curves.

(ii) Specialized packages, such as *PRINSAS* (Hinde, 2004 ▸), *McSAS* (Bressler *et al.*, 2015 ▸) and *MatSAS* (Rezaeyan *et al.*, 2021 ▸), which are typically developed by small teams or individual researchers. Though offering specialized capabilities, some of these tools can suffer from inconsistent coding practices and inadequate documentation, presenting challenges for new users who are unfamiliar with the original development framework.

Accessibility of (U)SAS data analysis is further hindered by reliance on proprietary software, with MATLAB (https://www.mathworks.com/products/matlab.html) and Igor (https://www.wavemetrics.com/) being the most prominent. While MATLAB is available through unrestricted academic licences, Igor only provides its users with a 30-day free trial, limiting the reach of packages developed through Igor. Most recently, *ScatterX* (Xie *et al.*, 2024 ▸) has emerged as a promising standalone program with a user-friendly graphical user interface (GUI); however, unclear input data format specifications and the lack of an English version hinder broader adoption.

Compounding these issues, existing literature on SAS software primarily focuses on mathematical and physical foundations rather than algorithmic implementations. Greater communication in software design would facilitate improvements by external contributors from outside the original team of developers, thus enhancing accuracy and efficiency. Well-documented implementations, alongside open-source code, would also aid troubleshooting when results appear questionable, helping to differentiate between measurement errors, fit parameter issues and algorithmic inconsistencies.

The polydisperse spherical pore (PDSP) model [equation (1) below; see also Radlinski (2006 ▸)] is widely used to extract structural information from porous materials, such as sedimentary rocks with broad pore size distributions and fractal characteristics (Radlinski *et al.*, 2000 ▸). This model converts SAS data from reciprocal space into a pore size distribution function *f*(*r*) in real space, while also solving for key parameters such as the total porosity and specific surface area. Although there exist numerous software packages for pore distribution calculations (*e.g.McSAS*, *Irena*), tools specifically designed for the PDSP model – particularly valuable for geoscientists, a growing subset of SANS users – are uncommon. Historically, *PRINSAS*, a program based on Microsoft *Excel*, was widely used for solving the PDSP model (Radlinski *et al.*, 2004*b* ▸; Bahadur *et al.*, 2015 ▸), but updates to *Excel* in 2008 rendered the program non-functional due to changes made in the solver function. Since then, the only available alternative has been *MatSAS*, a MATLAB-based script-driven program that inherits the limitations of MATLAB dependency and scripting-based workflows, posing challenges for users without a computing background.

We here introduce *PRINSAS 2.0*, an open-source stand­alone Python GUI program designed to fit the PDSP model to reduced one-dimensional SAS data, specifically addressing the needs of geoscientists and researchers studying porous systems. Tailored for users with limited coding expertise, *PRINSAS 2.0* prioritizes accessibility through an intuitive GUI and simplified input/output formats aligned with geological workflows. While optimized for ease of use, the program’s core computational engine, the fit_PDSP_model() function, is modularly designed to facilitate integration into broader Python-based analysis frameworks, ensuring flexibility for advanced users.

This paper presents the underlying mathematical background of the PDSP model. More details on the computational design and implementation are provided in Section S2 in the supporting information, allowing future enhancements to be more seamlessly incorporated. Comparison with fit results obtained from existing tools found in the literature, as well as validation of the software’s capabilities, was done using a range of modelled data sets. The Python implementation of the core fit_PDSP_model() function mirrors MATLAB’s computational logic, facilitating adaptation for MATLAB users into their own code base. Practical guidance on using the GUI, configuring inputs and interpreting results is provided in Section S3, with step-by-step instructions for users. *PRINSAS 2.0* and its source code are available for download from the *PRINSAS* GitHub repository (https://github.com/henry-pnhvu/PRINSAS-2.0) and will be continually supported to enhance usability and improve workflow efficiency for its users.

## 2. The PDSP model

For a two-phase system where the density is uniform and scale independent (*e.g.* homogeneous matrix and pores/air), the structure can be approximated as a collection of polydisperse spherical objects. The differential scattering cross section (dΣ/dΩ), spanning a sufficiently broad *Q* range (length scales) where *Q* = (4π/λ)sin(θ/2) (θ being the scattering angle and λ the wavelength of the incident radiation), can be described using the PDSP model (Hinde, 2004 ▸; Radlinski *et al.*, 2004*a* ▸),
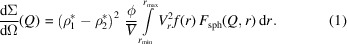
Here 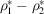 is the scattering contrast between the two phases [where 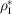 and 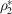 are the scattering length density (SLD) of the first and the second phase, respectively], ϕ is the total porosity (*i.e.* the ratio of pore volume to sample volume), 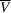 is the average pore volume, *V*_*r*_ is the pore volume corresponding to pores of radius *r* and *f*(*r*) is the probability density function (number-weighted distribution) of the pore sizes. [*r*_max_, *r*_min_] is the range of the examined feature sizes and corresponds to the investigated *Q* range; for fractal- and near-fractal-like scattering geological materials, it is assumed that *r*_max_ ≃ 2.5/*Q*_min_ and *r*_min_ ≃ 2.5/*Q*_max_ (Radliński *et al.*, 2000 ▸).

For spherical pores, the form factor *F*_sph_ is written as
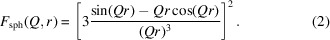


Equation (1) is derived from Debye’s law for two-phase systems (Melnichenko, 2016 ▸), where ϕ(1 − ϕ) is approximated as ϕ for systems with low porosity (<∼5%). For systems with higher porosity, equation (1) is written as
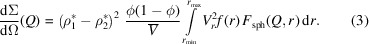


The continuous range of pore radius *r* in the number-weighted pore distribution *f*(*r*) in equation (3) can be divided into discrete *r*_*i*_ values, where *i* denotes the index (position) of each discrete value within the range. The scattering intensity of the overall (U)SAS profile can be numerically expressed as the sum of the scattering contributions from each *r*_*i*_ value as
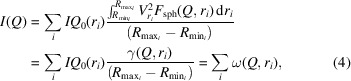
where 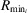 and 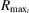 denote the limits of the continuous pore size distribution corresponding to the discrete pore radius value *r*_*i*_, 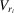 is the spherical volume corresponding to the pore radius *r*_*i*_, and *F*_sph_(*Q*, *r*_*i*_) is the spherical form factor corresponding to the current scattering vector magnitude *Q* and pore radius *r*_*i*_. *IQ*_0_(*r*_*i*_) is the scattering intensity at *Q* = 0 corresponding to *r*_*i*_ and has the form of
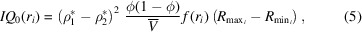
where the average pore volume 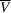 of the entire examined *Q* range is calculated as
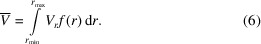


The PDSP model, which assumes that all pores are spherical, and the fitting result represent *a simplified approximation* of complex porous systems. Moreover, the PDSP model describes a dilute system of isolated, non-interacting spheres, which, taken strictly, implies the absence of connected porosity. This is inconsistent with (i) microscopy and modern tomography results (Cnudde & Boone, 2013 ▸), where real porous systems appear far more complex and interconnected than the model suggests, and (ii) fluid intrusion techniques, such as mercury intrusion porosimetry and gas sorption, which inherently rely on pore connectivity. Nevertheless, the PDSP model enables comparison between different samples or for a single sample under different experimental conditions and helps correlate SANS microstructural data with those obtained using other characterization methods, such as the aforementioned intrusion techniques..

## 3. Sample scattering data

**Figure 1:**
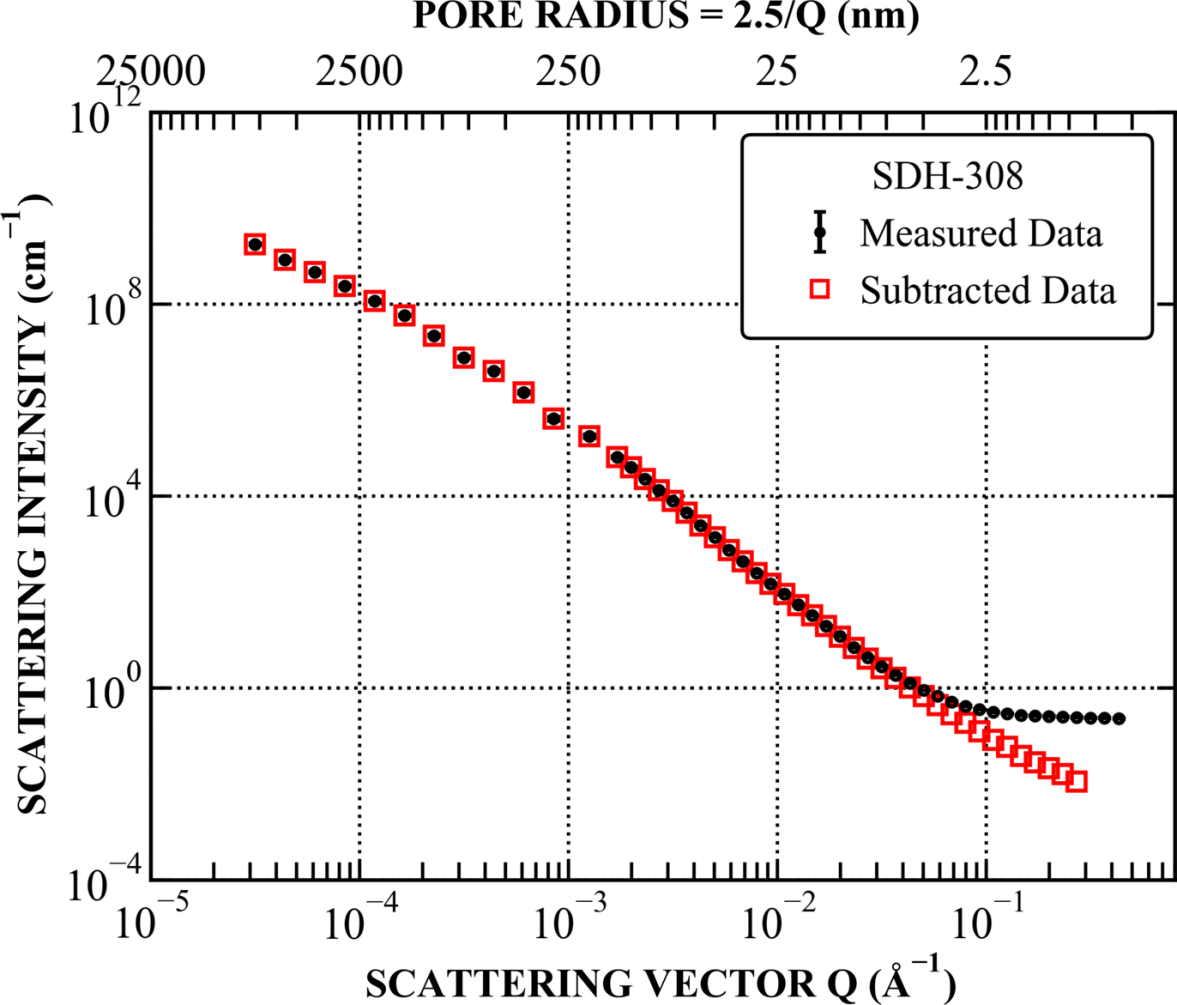
Combined SANS and USANS data for a New Albany Shale sample. Data after subtraction of the high-*Q* incoherent background are used to demonstrate the fitting procedure.

The combined small- and ultra-small-angle neutron scattering (SANS and USANS) data used in this study for demonstrating the fitting procedure of *PRINSAS 2.0* were obtained from a marginally mature Devonian New Albany Shale sample with a fractal pore size distribution, collected from a core drilled in Indiana, USA (Blach *et al.*, 2021 ▸). Scattering measurements were carried out on a thinned wafer sample using the Quokka and Kookaburra instruments at the Australian Nuclear Science and Technology Organisation (ANSTO) (Rehm *et al.*, 2018 ▸; Wood *et al.*, 2018 ▸), covering a combined *Q* range from 2 × 10^−5^ to 0.5 Å^−1^ (Fig. 1 ▸).

In addition to the experimental data, synthetic data with predefined pore size distributions were generated and tested using the software to verify its ability to give an accurate reconstruction of the original input pore size distributions. The software’s compatibility with diverse input formats was assessed by testing

(i) reduced one-dimensional data sets generated by instruments Quokka (SANS), Kookaburra (USANS) and Bilby [time-of-flight SANS; Sokolova *et al.* (2019 ▸)] at ANSTO, and beamlines D11 (SANS) and S18 (USANS) at the Institut Laue–Langevin (Lindner *et al.*, 1992 ▸; Kroupa *et al.*, 2000 ▸; Lindner & Schweins, 2010 ▸), and

(ii) additional synthetic data sets available in the *McSAS* GitHub repository (Pauw, 2015 ▸) with varying file structures, such as different header sizes and delimiters.

## 4. Fitting of the PDSP model in the core fitting function fit_PDSP_model()

The process of fitting the PDSP model to (U)SAS data follows the sequence of steps outlined below, with further details provided in the respective sections quoted:

(i) Estimate the *r* limits *r*_max_ and *r*_min_ of the result from the background-subtracted SAS data input, approximately as *r*_max_ = 2.5/*Q*_min_ and *r*_min_ = 2.5/*Q*_max_. The discrete radius *r*_*i*_ can be obtained by dividing the continuous *r* range between *r*_min_ and *r*_max_ into equal intervals on a logarithmic scale (Section 4.1 and Section S2.1).

(ii) Form a matrix of all possible *Q* and *r*_*i*_ values and numerically calculate γ(*Q*, *r*_*i*_) in equation (4) (Section S2.2); the list of *Q* values is obtained directly from the input SAS data.

(iii) Estimate the starting values of *IQ*_0_(*r*_*i*_), [*IQ*_0_(*r*_*i*_)]_start_, prior to the fitting procedure, done by assuming that each measured *I*(*Q*) value consists solely of the scattering contribution at *r*_*i*_ = 2.5/*Q* (Section 4.2).

(iv) Iteratively adjust the individual *IQ*_0_(*r*_*i*_) values, such that the sum of the squares of the residuals between *I*(*Q*)_calc_ [calculated using equation (4)] and *I*(*Q*)_measure_, weighted by the measurement error, reaches a minimum (Section 4.3).

(v) Use the thus-fitted *IQ*_0_(*r*_*i*_) to calculate the dependent parameters, such as the porosity, pore volume and other structural variables (Section 4.6).

### 4.1. Determining the *r* range and *r*_*i*_ values

The *r* range [*r*_min_, *r*_max_] of the fitting results, corresponding to the *Q* range of the input (U)SAS data, is determined as *r*_min_ = 2.5/*Q*_max_ and *r*_max_ = 2.5/*Q*_min_. A set of *r*_*i*_ values is generated within this [*r*_min_, *r*_max_] interval by discretizing the continuous *r* range. The density of these values, which determines how finely the pore size distribution is subdivided, is controlled by specifying the number of *r*_*i*_ values per decade of *r*.

For optimal fit results, the number of *r*_*i*_ values within the *r* limits should be chosen (by users) such that the total count of *r*_*i*_ remains less than half the number of (U)SAS data points. This constraint serves two purposes:

(i) limiting the number of *r*_*i*_ values prevents overfitting, where the model becomes overly sensitive to experimental noise rather than capturing the true structural characteristics of the sample, while

(ii) ensuring a sufficient number of *r*_*i*_ values allows for an accurate and reliable representation of the sample’s pore size distribution.

### 4.2. Initialization of *IQ*_0*i*_ values

Prior to the fitting procedure, the determination of the starting value of *IQ*_0_(*r*_*i*_), [*IQ*_0_(*r*_*i*_)]_start_, is required. This process serves as the foundation for subsequent iterative optimization of *IQ*_0_(*r*_*i*_), ensuring that the model begins with a reasonable approximation of the experimental data, forming a ‘bias’ for the fitting procedure.

**Figure 2:**
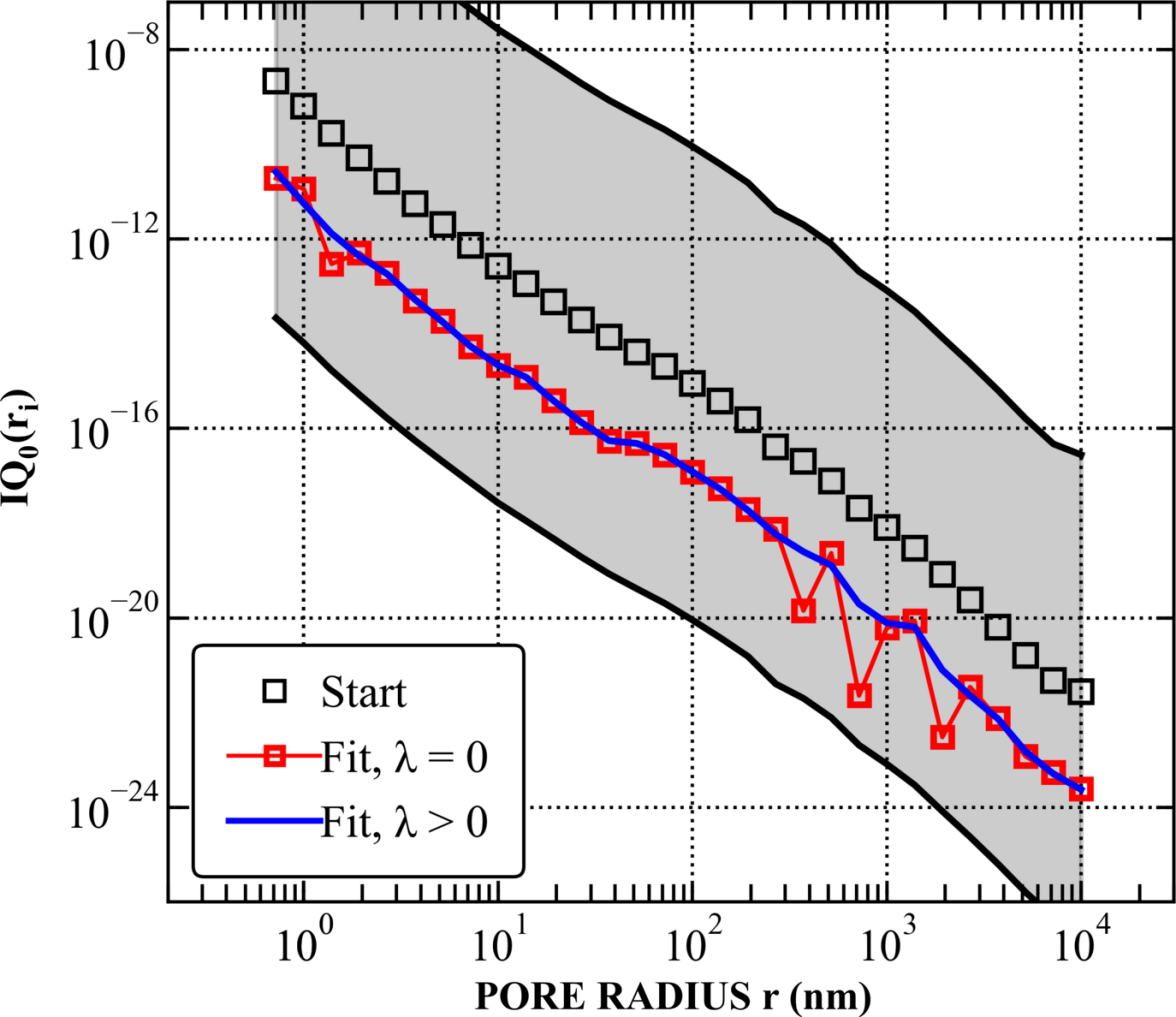
Comparison between the starting *IQ*_0_(*r*_*i*_) values prior to the optimization process and the fitted *IQ*_0_(*r*_*i*_) values, with and without the smoothing factor λ. Upper and lower solid black lines, along with shaded area in between, indicate the permissible fitting range for *IQ*_0_(*r*_*i*_), spanning five orders of magnitude above and below the starting values. For clarity, the fit results at λ = 0 and λ > 0 are divided by a factor of 100.

**Figure 3:**
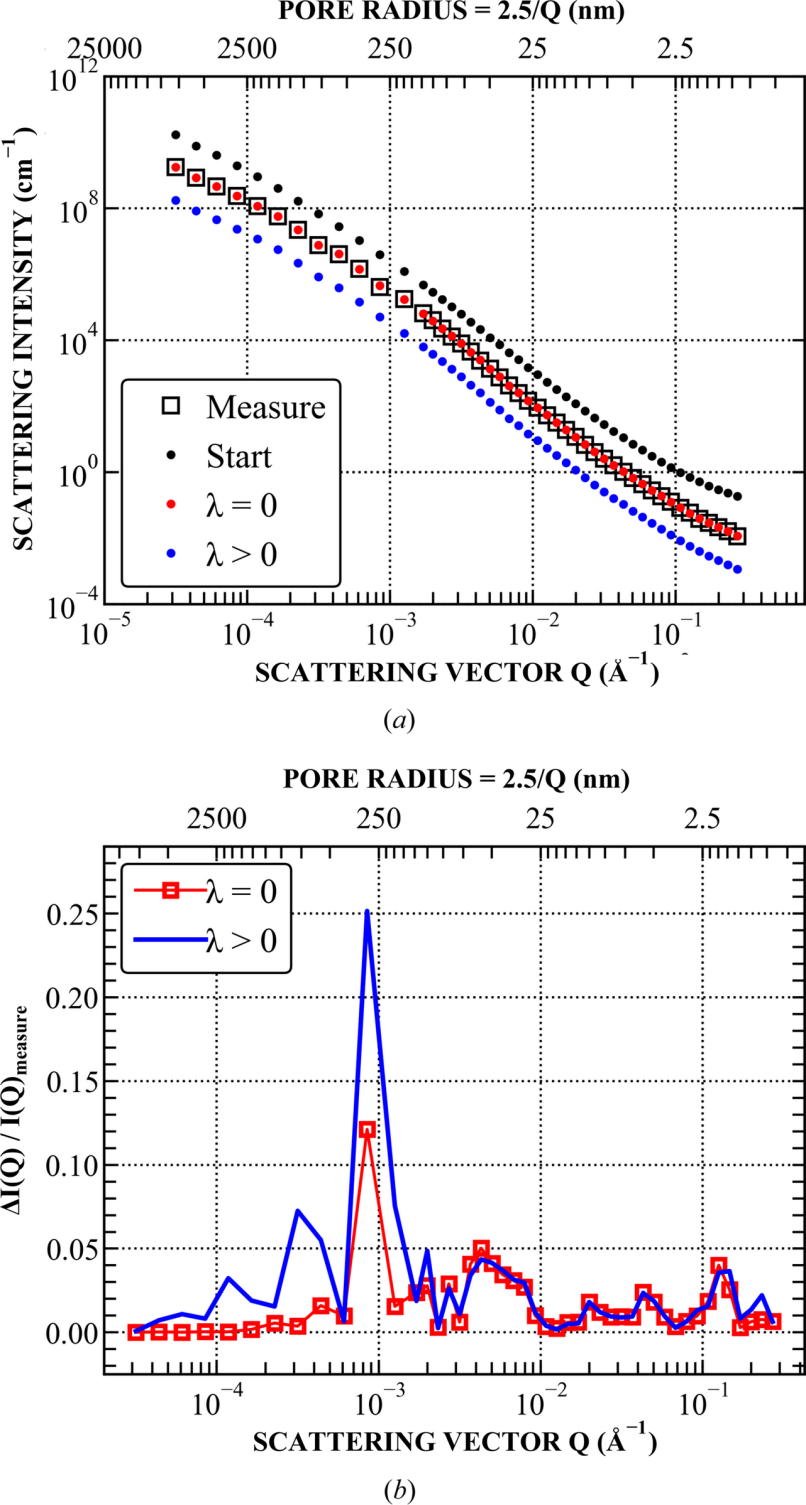
(*a*) Comparison between (i) the measured *I*(*Q*) values with background subtracted, (ii) the starting *I*(*Q*) values prior to the fitting procedure, and (iii) the fitted *I*(*Q*) values with and without the smoothing factor λ. The starting and fitted values were calculated using equation (4) and their corresponding *IQ*_0_(*r*_*i*_) values shown in Fig. 2; for clarity, the starting *I*(*Q*) values and fitted *I*(*Q*) values with λ > 0 are multiplied by factors of 10 and 0.1, respectively. (*b*) Deviation of the fitted *I*(*Q*) values (with and without smoothing) from the measured *I*(*Q*) data.

This initial estimate is obtained by assuming that the scattering intensity at a given *Q* originates entirely from pores with radii *r*_*i*_ = 2.5/*Q*, rather than the up to 66% contribution in reality (Radlinski *et al.*, 2004*a* ▸). Under this assumption, [*IQ*_0_(*r*_*i*_)]_start_ can then be estimated from equation (4) as
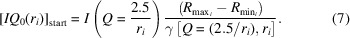
Since there are many possible solutions for the nonlinear fit of *IQ*_0_(*r*_*i*_), starting with a value closest to the most physically probable one helps eliminate less realistic alternatives. In systems with fewer distinct features – commonly encountered in geological materials – the initial estimate often closely approximates the final solution, as demonstrated in Figs. 2 and 3.

### 4.3. The iterative fitting procedure

Once the [*IQ*_0_(*r*_*i*_)]_start_ values have been determined, they are substituted into equation (4) alongside the computed values of γ(*Q*, *r*_*i*_) to generate a calculated scattering intensity profile *I*(*Q*)_calc_. The fitting procedure aims to minimize the misfit between the calculated and measured intensity *I*(*Q*)_measure_, defined as
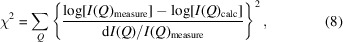
where d*I*(*Q*) is the measurement error associated with each *I*(*Q*) value.

Equation (8) is adapted from equation (4) of Potton *et al.* (1988 ▸), modified to account for the power-law nature of the scattering signal. This transforms the fitting problem into a more balanced and approximately linear one, while retaining the original weighting by d*I*(*Q*). To increase linearity further, prior to the calculation of χ^2^, all of *I*(*Q*)_calc_, *I*(*Q*)_measure_ and d*I*(*Q*) in equation (8) are multiplied by *Q*^−*n*^, where *n* is the linear slope fitted through the measured scattering data on a log–log plot.

In addition, to ensure that the fitting process properly accounts for variations in *IQ*_0_(*r*_*i*_) values across all scales, the variation of *IQ*_0_(*r*_*i*_) during the optimization process is performed on a logarithmic scale. This approach accounts for the power-law distribution of pore sizes common in geological systems, where the intensity contributed by smaller pores is significantly lower than that from larger ones. This prevents the disproportionate weighting of larger pores, which would otherwise skew the results.

The fitting procedure commences by cycling through the different values of *IQ*_0_(*r*_*i*_) over the entire *r*_*i*_ range, such that χ^2^ reaches a minimum. This optimization procedure is performed automatically using the minimize() function in the Python *SciPy* library (The SciPy community, 2024 ▸).

The allowed range of fitted values *IQ*_0_(*r*_*i*_)_fit_ is set arbitrarily to span five orders of magnitude above and below the initial [*IQ*_0_(*r*_*i*_)]_start_ estimates (Fig. 2). This range is chosen to provide sufficient flexibility for the optimization algorithm to explore potential solutions while maintaining numerical stability. A narrower range could lead to local minima trapping the optimization, whereas an excessively wide range might introduce instability or unrealistic solutions.

### 4.4. Smoothing of the fit result

An additional smoothing procedure can be applied to improve the fit quality further and reduce the noise level in the fitted values of *IQ*_0_(*r*_*i*_)_fit_. This helps minimize artificial variations caused by overfitting to minor inconsistencies (noise) in the measured data. To achieve this, a simple regularization term is introduced into the optimization equation (8), resulting in
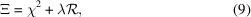
where λ is a scaling factor. The regularization term 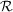 is defined as the sum of squared gradients between adjacent points in the fit result. This regularization term is chosen under the assumption that the transition in pore size distribution at different *r* values in a geological system is smooth and continuous, taking the form of
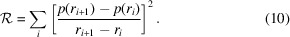
Here, the normalized pore size distribution *p*(*r*_*i*_) is obtained by multiplying *IQ*_0_(*r*_*i*_) by 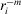, where *m* is the linear slope fitted through the *IQ*_0_(*r*_*i*_) versus *r* plot on a log–log scale at the current fit iteration. This transformation linearizes the power-law distribution of *IQ*_0_(*r*_*i*_), ensuring a more balanced weighting across the *r*_*i*_ values.

Smoothing is introduced by altering the scaling factor λ in equation (9) such that the regularization term 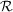 carries greater weight in the calculation of Ξ, causing the fitting algorithm to prioritize minimizing the value of 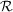 over χ^2^. As 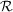 corresponds to the sum of squared gradients in the fitted result, a lower 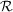 value results in a smoother *IQ*_0_(*r*_*i*_) curve. However, this smoothing comes potentially at the cost of a higher χ^2^ value, reflecting greater deviation between *I*(*Q*)_calc_ and *I*(*Q*)_measure_.

Comparisons between the stated values [*IQ*_0_(*r*_*i*_)]_start_ (determined in Section 4.2) and the fitted values *IQ*_0_(*r*_*i*_)_fit_, both with and without the smoothing factor λ, are demonstrated in Figs. 2 ▸ and 3 ▸. The results indicate that the fitted *IQ*_0_(*r*_*i*_)_fit_ values in the *r* range between 200 and 2000 nm are the noisiest, corresponding to *Q* between 10^−4^ and 10^−3^ Å^−1^. This *Q* range primarily consists of USANS data, which often exhibit structure-unrelated fluctuations due to (i) shifts in the Si crystal used for USANS experiments caused by temperature changes, (ii) weaker scattering at large USANS angles and (iii) numerical artefacts introduced during the USANS desmearing process. Consequently, the smoothing effect is most prominent in this region, evident from the significantly lower fluctuation level of *IQ*_0_(*r*_*i*_)_fit_ and the increased deviation between *I*(*Q*)_calc_ and *I*(*Q*)_measure_ for λ > 0.

### 4.5. Error estimation

The error in the fit results is approximated using equation (7), with *I*(*Q*) being replaced by σ(*Q*). Here, σ(*Q*) at each *Q* value is defined as the greater of (i) the relative deviation between *I*(*Q*)_fit_ and *I*(*Q*)_measure_ and (ii) the measurement error d*I*(*Q*), with both expressed as percentages of *I*(*Q*)_measure_. Using this approach, the error in the pore size distribution at each *r*_*i*_ is assumed to inherit the uncertainty from the scattering intensity at the corresponding *Q* = 2.5/*r*_*i*_. Note that this error estimate reflects only the numerical quality of the fit and does not serve as a formal confidence interval for *IQ*_0_(*r*_*i*_)_fit_. It also does not account for measurement artefacts, such as those introduced by USANS desmearing exceeding experimental errors, or errors at large *Q* values resulting from background subtraction.

### 4.6. Calculation of sample properties from the PDSP fit result

Once the values of *IQ*_0*i*_ are obtained, the remaining physical properties of the measured sample can be calculated, including the probability density function of the pore sizes (also known as the number-weighted pore size distribution) *f*(*r*), porosity ϕ, average pore volume 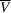, specific surface area (SSA) and differential pore volume distribution d*V*/d*r*.

Since
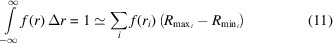
for a continuous function of the pore distribution *f*(*r*) with respect to *r*, the discrete *f*(*r*_*i*_) values can be estimated, deriving from equation (5)
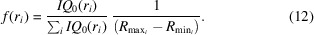


Next, the average pore volume can be calculated as
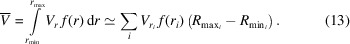


The value of the porosity ϕ can then be determined by substituting the result of *f*(*r*_*i*_) and 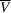 into equation (5), given a known contrast. Since equation (5) is symmetric with respect to phase 1 (with volume fraction ϕ) and phase 2 [with volume fraction (1 − ϕ)], the value of *IQ*_0*i*_ will not change if each phase is replaced by its counterpart with the corresponding volume fraction (Babinet, 1837 ▸). To determine accurately the volume fractions of the solid matrix and void from the PDSP model, independently obtained structural information about the measured sample is required. This may include data from helium porosimeters for systems with high percentages of accessible porosity relative to total porosity.

The SSA, determined using a probe with radius *R*, is defined as the sum of the SSAs of all pores with radii larger than *R* and is estimated as
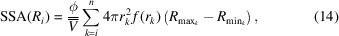
where *r*_*k*_ < *r*_*k*+1_.

Finally, the differential pore volume per unit weight is calculated as
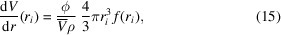
where ρ is the matrix average bulk density, determined as *m*_sample_/*V*_sample_.

Importantly, the porosity ϕ and its dependent parameters SSA(*R*) and d*V*/d*r* determined from the PDSP model are directly calculated from (and therefore are functions of) the SLD and absolute scattering intensity. Hence, obtaining reliable values for these fitted parameters requires accurate determination of both the SLD and the absolute intensity. However, this is not always achievable, especially for powdered samples.

## 5. Assessing the capabilities and limitations of the fitting algorithm

To assess the reliability of the fitting algorithm, a series of simulated SAS profiles were generated using different predefined profiles of *IQ*_0_(*r*_*i*_) called *IQ*_0_(*r*_*i*_)_sim_. These *IQ*_0_(*r*_*i*_)_sim_ profiles include (i) an ideal power-law size distribution with and without the addition of random noise, and (ii) a power-law size distribution with the addition or subtraction of monodispersed and bidispersed pore components. The uneven *Q* spacing in the simulated *I*(*Q*) profiles was chosen to reflect experimental data, where SAS exhibits much higher point density than USAS.

The fit results of these modelled data sets, performed without any smoothing, were then compared with the fit results acquired using

(*a*) the maximum entropy algorithm (called *MaxEnt*), as used in *Irena* and *GSAS-II*, adapted from *GSAS-II*’s source code (Advanced Photon Source, 2024 ▸), with a maximum iteration number of 10^4^, and

(*b*) Monte Carlo simulation, obtained directly from *McSAS* (Bressler *et al.*, 2015 ▸), with a maximum of 10^6^ iterations.

**Figure 4:**
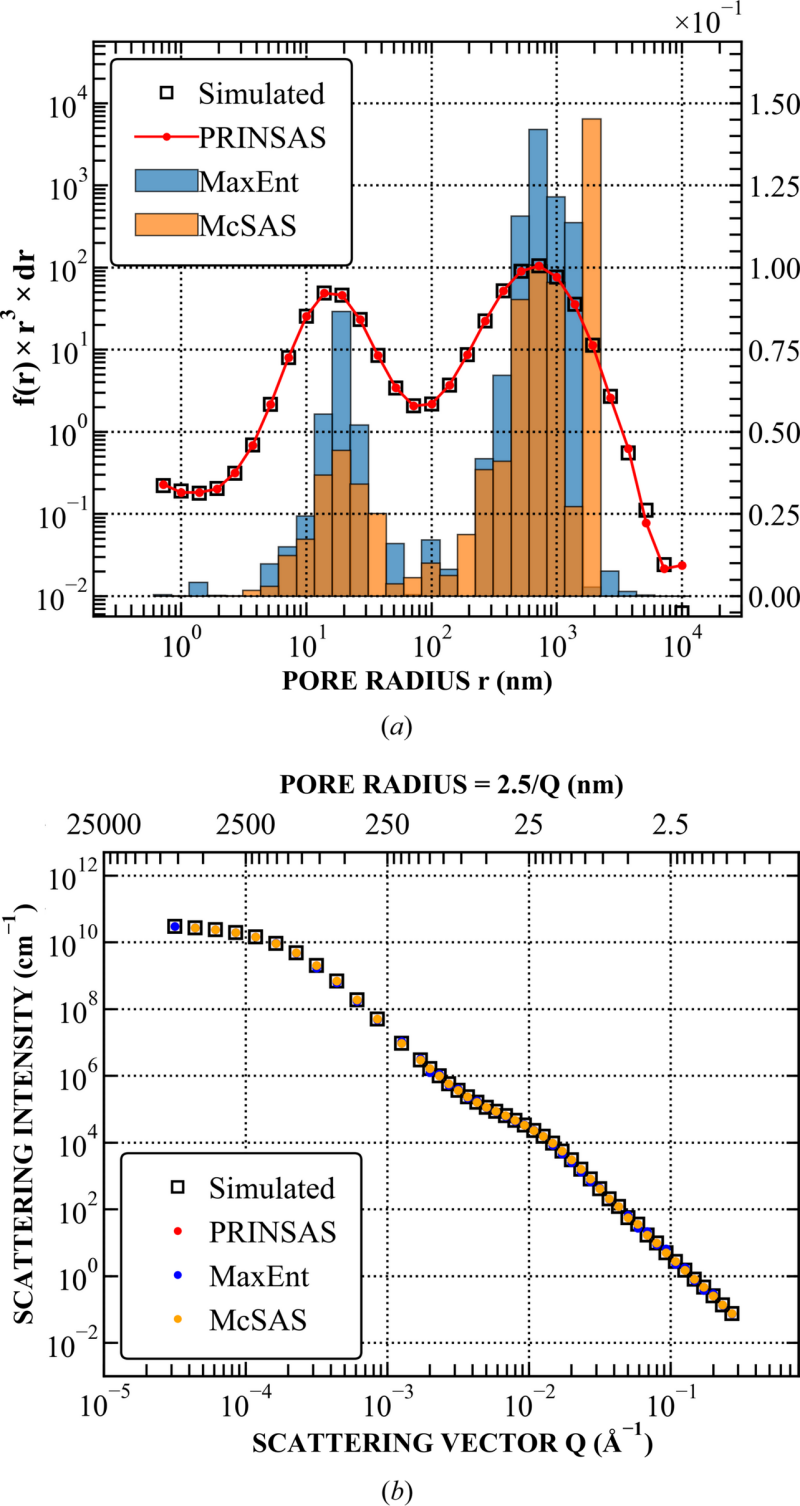
Fitting of the PDSP model to a power-law pore network superimposed with a bidisperse pore distribution. (*a*) Comparison between the simulated and fitted pore distributions. Simulated data and *PRINSAS* results are plotted on a log scale on the left-hand axis, whereas *MaxEnt* and *McSAS* results are plotted on a linear scale on the right-hand axis. (*b*) Comparison between the corresponding simulated and fitted *I*(*Q*).

**Figure 5:**
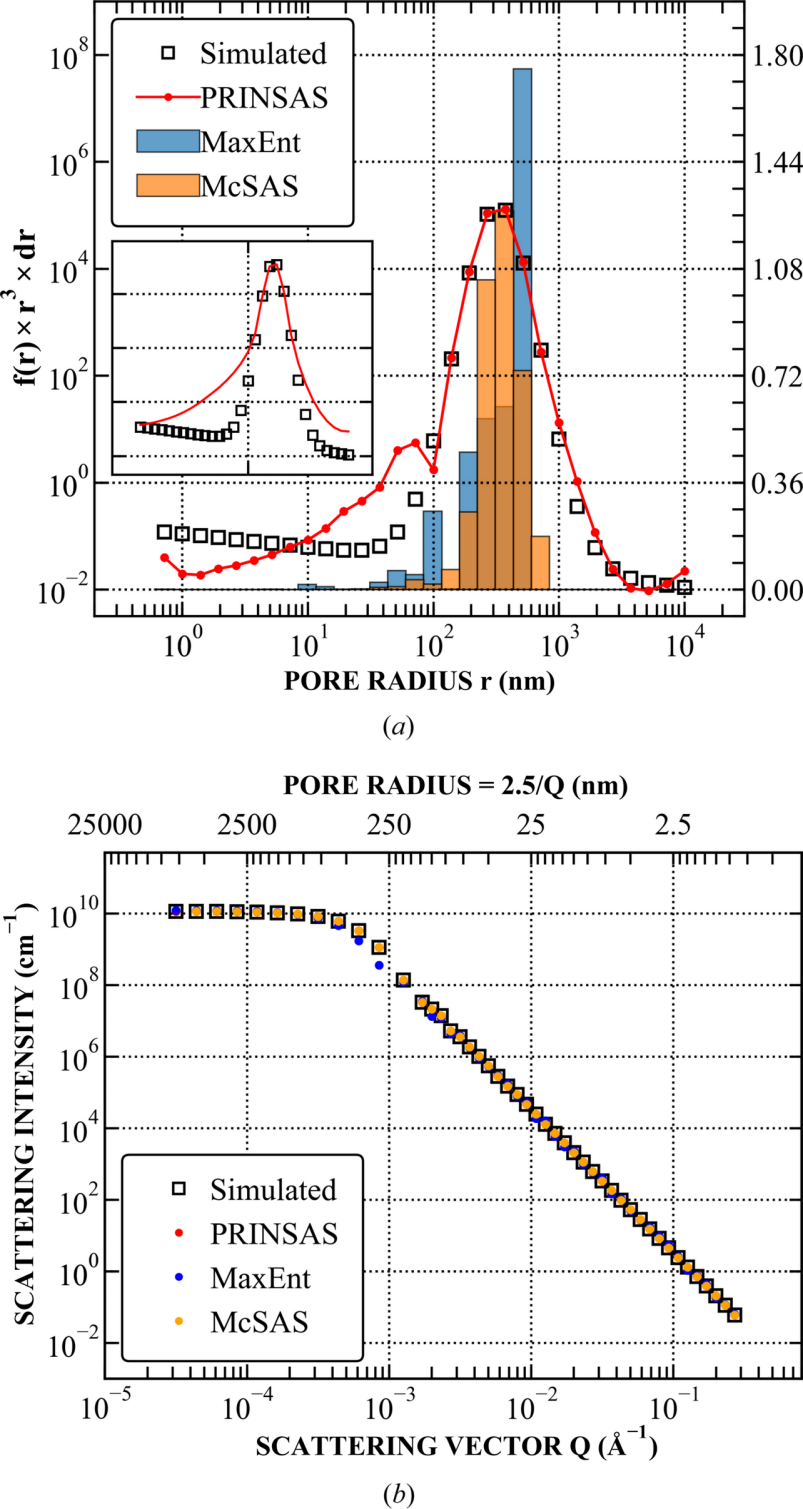
Fitting of the PDSP model to a power-law pore network superimposed with a sharp monodispersed pore distribution. (*a*) Comparison between the simulated and fitted pore distributions. Simulated data and *PRINSAS* results are plotted on a log scale (left-hand axis), whereas *MaxEnt* and *McSAS* results are plotted on a linear scale (right-hand axis). The inset shows the *PRINSAS* fit result with a smoothing factor λ > 0. (*b*) Comparison between the corresponding simulated and fitted *I*(*Q*).

**Figure 6:**
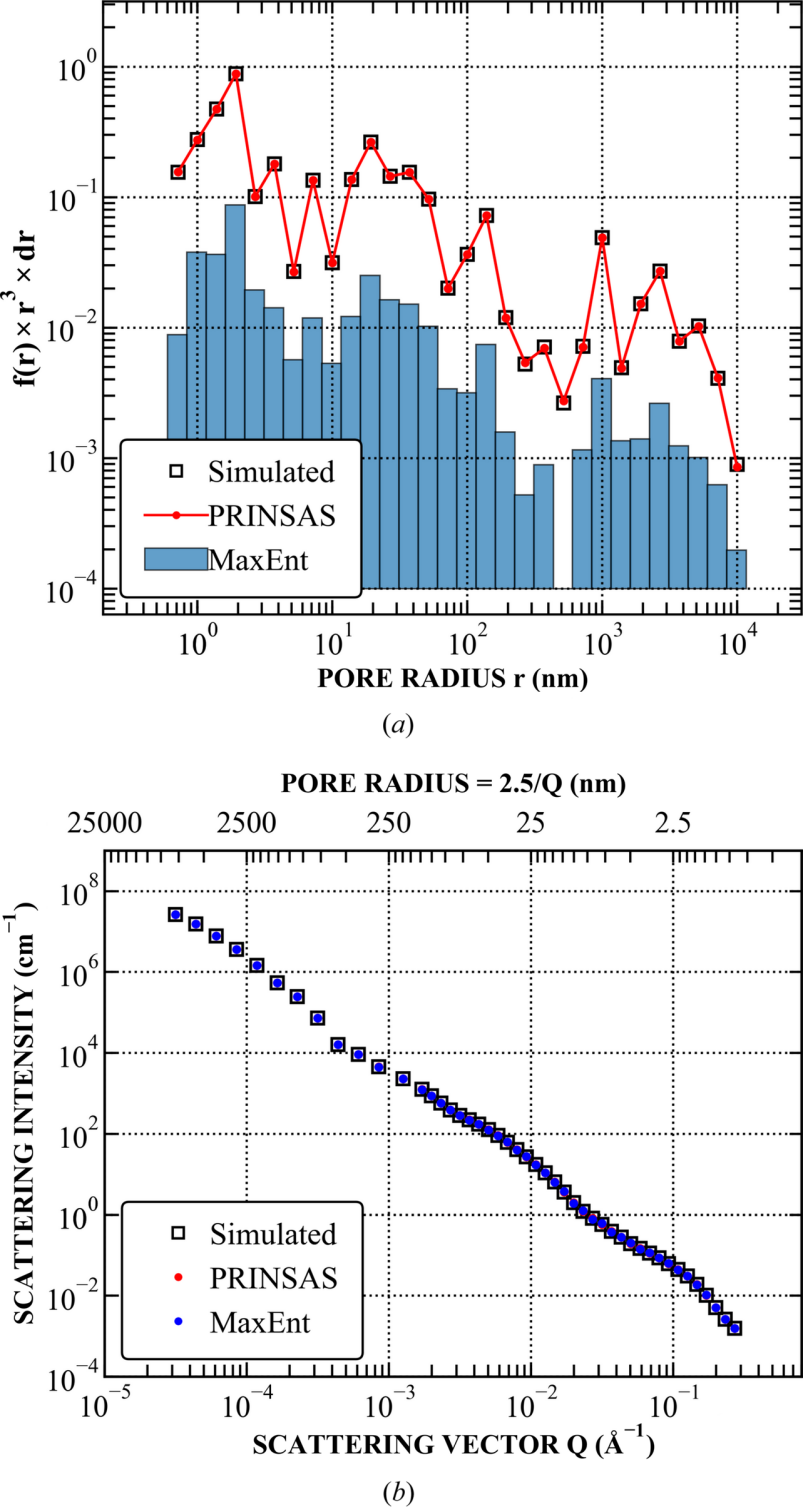
Fitting of the PDSP model to a power-law pore network with the addition of random noise values. (*a*) Comparison between the simulated and fitted pore distributions. *McSAS* fit results could not be obtained after 10^6^ iterations. (*b*) Comparison between the corresponding simulated and fitted *I*(*Q*).

**Figure 7:**
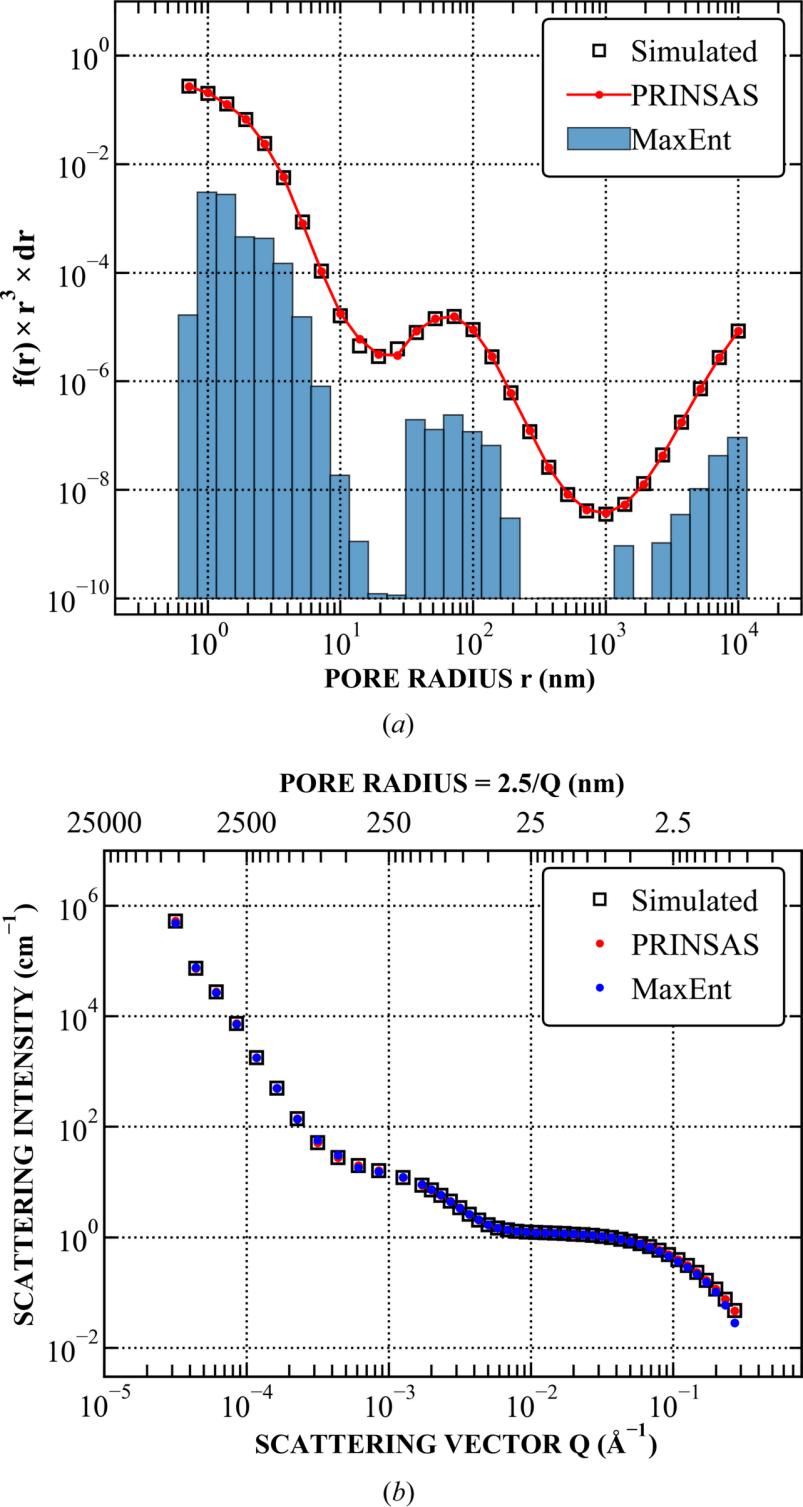
Fitting of the PDSP model to a power-law pore network with a bidisperse pore distribution subtracted to introduce two valleys in the data; such a system does not have real-life analogues and is used here to assess the software’s stability. (*a*) Comparison between the simulated and fitted pore distributions. *McSAS* fit results could not be obtained after 10^6^ iterations. (*b*) Comparison between the corresponding simulated and fitted *I*(*Q*).

Both methods were run using an identical pore size range and binning to *PRINSAS*. Representative examples are shown in Figs. 4–7, with the remaining test cases shown in Section S1. Results from *MaxEnt* and *McSAS* are displayed on either linear or logarithmic scales, depending on which best represents the fit.

For structures with broad but distinct features, *PRINSAS* can reliably reconstruct the input volume-weighted size distribution, with only minor discrepancies near the edges of the *r* range, and produces results comparable to those from *MaxEnt* and *McSAS* (Fig. 4 ▸). However, in cases involving SAS data with sharp features (Fig. 5 ▸), *PRINSAS*, while accurately capturing the position and magnitude of the dominant peak, introduces an artificial smaller feature on the left-hand side of the dominant peak, which does not exist in *IQ*_0_(*r*_*i*_)_sim_.

We postulate that this artefact arises from the large intensity difference between the peak and its surrounding regions. In cases where the scattering intensity is dominated by sharp features, these features primarily influence the overall scattering profile. As a result, beyond a certain threshold difference in pore concentration between the dominant-size feature and its surroundings, contributions from non-dominant pores become negligible. Consequently, the convergence of the fitted *I*(*Q*) curve to the simulated *I*(*Q*) in the *Q* regions surrounding the peak occurs without requiring a perfect match between *IQ*_0_(*r*_*i*_)_fit_ and the original *IQ*_0_(*r*_*i*_)_sim_. Introducing a smoothing factor suppresses this artefact while preserving the dominant structural feature [inset of Fig. 5 ▸(*a*)]; nevertheless, mismatches in the regions surrounding the sharp peak remain unresolved.

The high discrepancy in concentration across different *r* values affecting the fit results is also evident in Fig. S4, where the fitting algorithm overestimates the density at a sharp local minimum in the middle of the *IQ*_0_(*r*_*i*_) profile. There appears to be no clear threshold for peak sharpness that triggers significant discrepancies between *IQ*_0_(*r*_*i*_)_fit_ and *IQ*_0_(*r*_*i*_)_sim_. Consequently, when applying the PDSP model to systems with strongly preferential pore size distributions, the fit results are reliable for identifying the sizes of the dominant pores and their corresponding concentration and SSA values. The fitted results of the underlying structure, on the other hand, should be interpreted with caution.

For systems consisting of non-distinct pore features (Fig. 6 ▸), commonly encountered in geological materials, or those lacking clear Gaussian-like combinations of pore populations (Fig. 7 ▸), we could not obtain a satisfactory fit using *McSAS*, while both *PRINSAS* and *MaxEnt* show good agreement with each other and are capable of accurately reconstructing the original pore size distribution.

## 6. Conclusion

*PRINSAS 2.0* delivers critical updates to the original 2004 software by transitioning from proprietary Microsoft *Excel* and *Access* frameworks to an open-source Python platform. Redesigned explicitly for geoscientists analysing porous mater­ials, the new software streamlines the polydisperse spherical pore model fitting process via an intuitive graphical user interface. Its modular architecture allows further development of additional application-specific features, including seamless integration of the core fitting function fit_PDSP_model() into other Python-based SAS analysis tools.

This paper outlines the scientific background and design rationale underpinning the PDSP fitting procedure, facilitating future enhancements and integration with other software packages. Validation against synthetic data sets and experimental SANS/USANS profiles, along with comparisons with other established tools, demonstrates the software’s robust performance in reconstructing complex pore size distributions. Additionally, its compatibility with diverse input data formats ensures broad applicability.

By prioritizing accessibility without compromising analytical precision, *PRINSAS 2.0* allows non-specialist users to employ neutron scattering data more effectively for advanced nanoscale characterization of porous systems. Future software developments may extend the model to incorporate non-spherical pore geometries, further enhancing its utility across interdisciplinary research domains.

## Supplementary Material

Additional results and user instructions. DOI: 10.1107/S1600576725004315/vb5093sup1.pdf
