# Supplementary material for: PRINSAS 2.0: a Python-based graphical user interface tool for fitting polydisperse spherical pore models in small-angle scattering analysis of porous materials
Source: J Appl Crystallogr. 2025 Jul 2;58(Pt 4):1486–95. doi: 10.1107/S1600576725004315 (PMC12321026; doi:10.1107/S1600576725004315)
Supplement: Supplementary file 1 [file j-58-01486-sup1.pdf]

JOURNAL OF
APPLIED
CRYSTALLOGRAPHY

Volume 58 (2025)

Supporting information for article:

PRINSAS 2.0: a Python-based graphical user interface tool for fitting polydisperse spherical pore models in small-angle scattering analysis of porous materials

Phung Nhu Hao Vu, Andrzej P. Radlinski, Tomasz Blach, John Daniels and Klaus Regenauer-Lieb

Section S1 Fit result of PDSP model to simulated SANS profiles

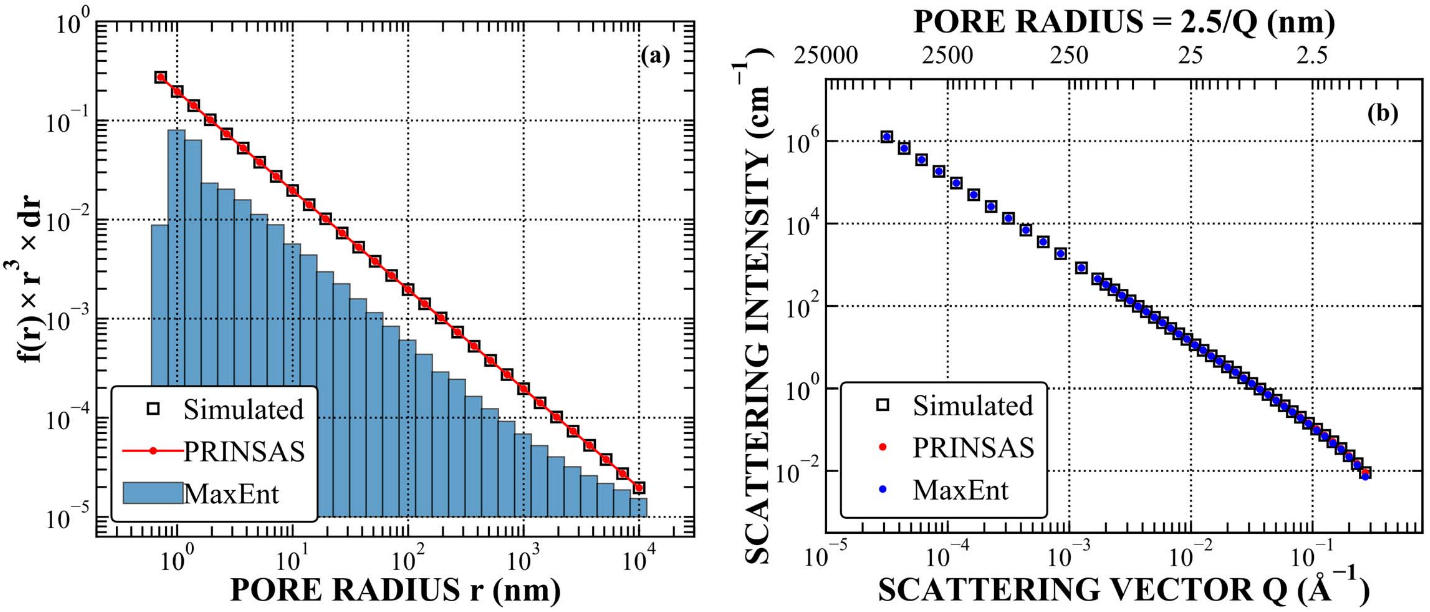

Figure S1 Fitting of the PDSP model to an ideal power-law pore network. (a) Comparison between the simulated and fitted pore distribution; McSAS fit results could not be obtained after 10^6 iterations. (b) Comparison between the corresponding simulated and fitted $I(Q)$.

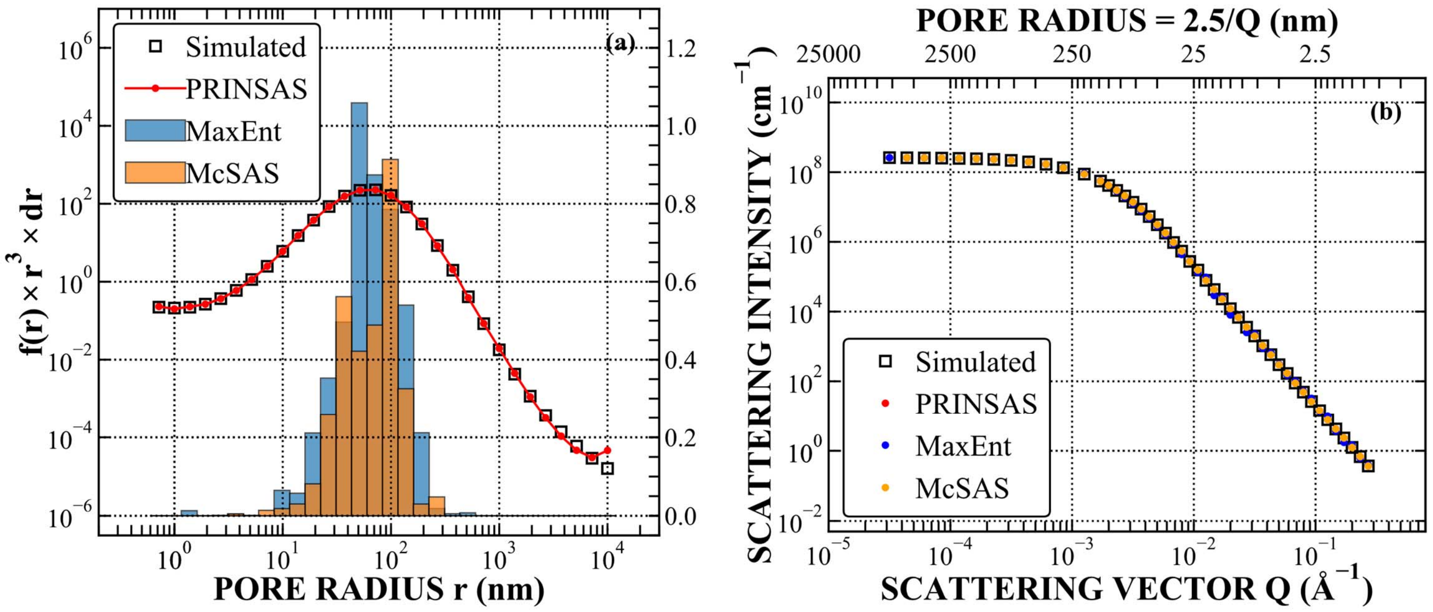

Figure S2 Fitting of the PDSP model to a power-law pore network superimposed with a monodispersed pore distribution. (a) Comparison between the simulated and fitted pore distribution; simulated data and PRINSAS result are plotted on a log-scale on the left axis, whereas MaxEnt and McSAS results are plotted on a linear scale on the right axis. (b) Comparison between the corresponding simulated and fitted $I(Q)$.

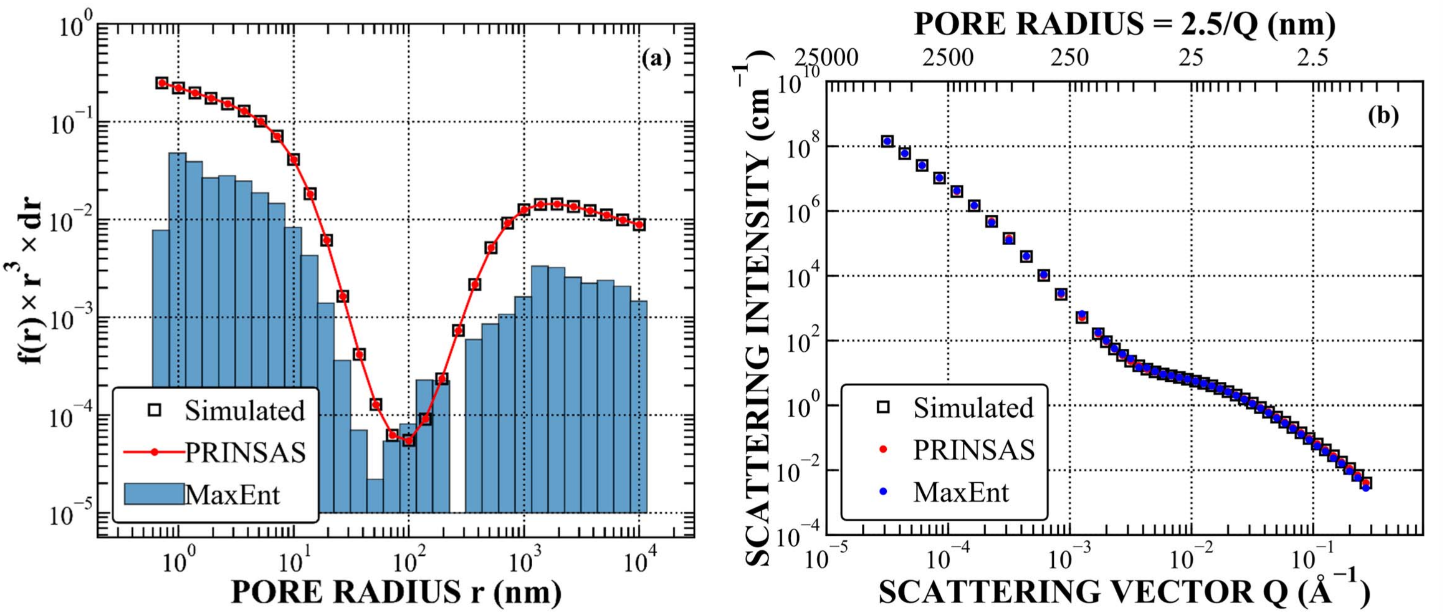

Figure S3 Fitting of the PDSP model to an ideal power-law pore network with a monodispersed pore distribution subtracted to introduce a dip in the data; such a system does not have real-life analogues and is used here to assess the software's stability. (a) Comparison between the simulated and fitted pore distribution; simulated data and PRINSAS results are plotted on a log-scale on the left axis, whereas MaxEnt and McSAS results are plotted on a linear scale on the right axis; McSAS fit results could not be obtained after 10^6 iterations. (b) Comparison between the corresponding simulated and fitted $I(Q)$.

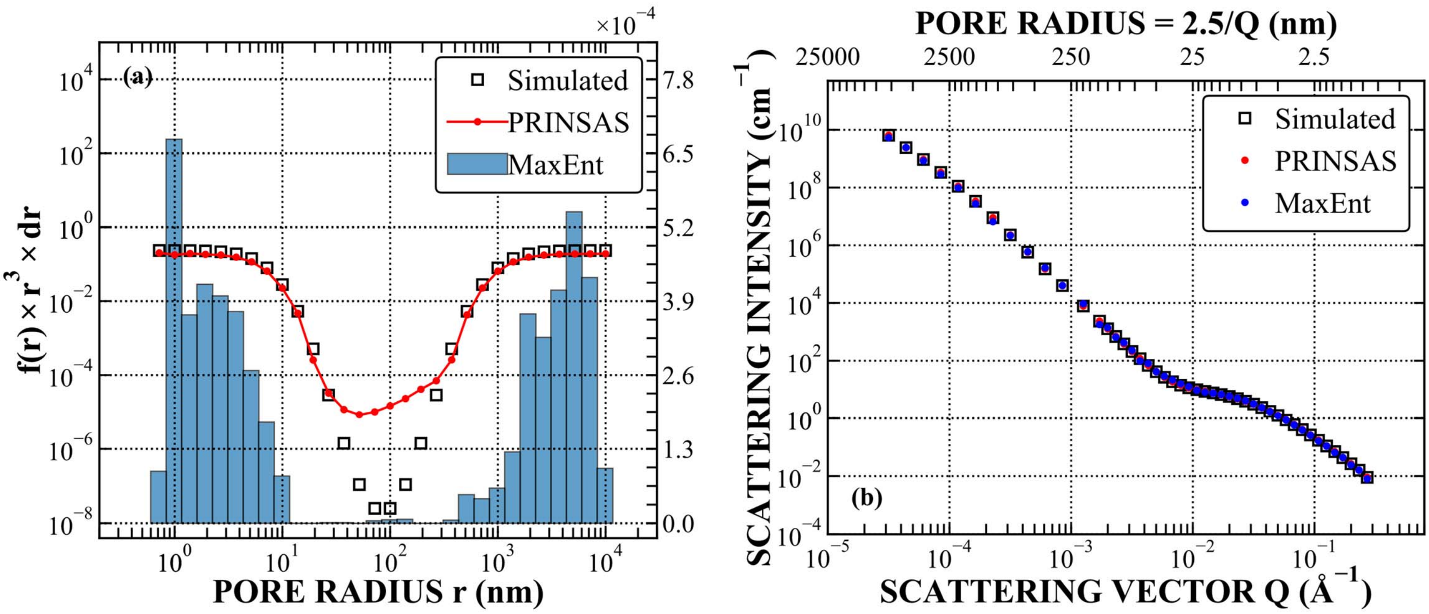

Figure S4 Fitting of the PDSP model to an ideal power-law pore network with a sharp monodispersed pore distribution subtracted to introduce a valley in the data; such a system does not have real-life analogues and is used here to assess the software's stability. (a) Comparison between the simulated and and fitted pore distribution; simulated data and PRINSAS results are plotted on the log-scale on the left axis, whereas MaxEnt and McSAS results are plotted on the linear scale on the right axis; McSAS fit results could not be obtained after 10^6 iterations. (b) Comparison between the corresponding simulated and fitted $I(Q)$.

Section S2 Numerical implementation of the software

Section S2.1 Creation and binning of r_i values

In addition to the steps outlined in Section 4.1, an extra procedure is performed to ensure that the obtained equally-spaced r_i values coincide with the rounded decade markers on the log-scale axis (*e.g.* $r_i = 10^y$ nm, with y being an integer). This alignment helps maintain consistency of the r range across different input (U)SAS profiles, making comparisons more reliable regardless of possible slight variations in the examined Q ranges.

The process of obtaining the r_{min}, r_{max}, and r_i values is illustrated in Fig. S5, starting with the determination of $(r_{min})_{calc} = 2.5/Q_{max}$ and $(r_{max})_{calc} = 2.5/Q_{min}$ from the given Q range. Next, logarithmically spaced decade markers are divided into equal intervals with the spacing between each r_i dictated by the specified number of points per decade. Finally, a continuous set of r_i values is then

selected such that (i) the minimum chosen value—r_{min}—is the largest r_i value smaller than $(r_{min})_{calc}$, and (ii) the maximum chosen value—r_{max}—is the smallest r_i value larger than $(r_{max})_{calc}$.

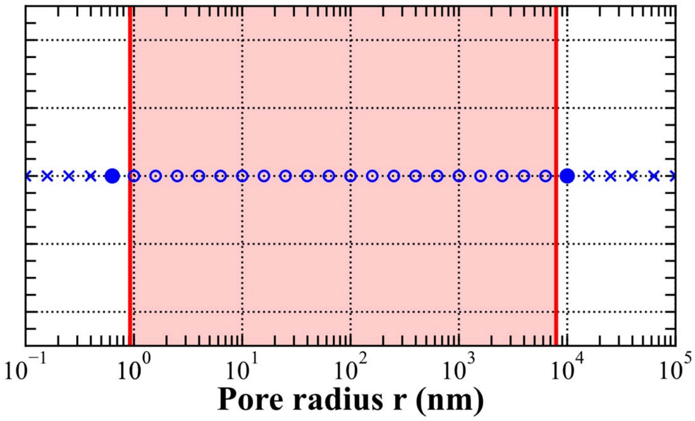

Figure S5 Demonstration of the determination of r_i values. Blue makers represent all the available r values between the r range of 0.1 and 10^5 nm, with a point density of five r_i values per decade. Two vertical red lines represent $(r_{min})_{calc}$ and $(r_{max})_{calc}$, calculated as $(r_{min})_{calc} = 2.5/Q_{max}$ and $(r_{max})_{calc} = 2.5/Q_{min}$. Shaded area denotes the r range covered by $(r_{min})_{calc}$ and $(r_{max})_{calc}$,. Circular markers are the r_i values selected for fitting the PDSP model, whereas crosses indicate non-selected values. Solid circular markers indicate the selected minimum and the maximum r_i value, r_{min} and r_{max}.

Section S2.2 Calculation of the integral $\gamma(Q, r_i)$

$\gamma(Q, r_i)$ in equation (4) is numerically calculated using the mid-point method, which approximates the integral of a function over a continuous interval by dividing the interval into smaller, equally spaced sub-intervals. The integral is then computed by (i) evaluating the function at the midpoint of each sub-interval, (ii) multiplying the function value at each midpoint by the width of the sub-interval, and (iii) summing these products across all sub-intervals to obtain the total approximation of the integral. This method is chosen for its relatively lower approximation error compared to other numerical integration techniques such as the trapezoidal rule.

Assuming that $V_r^2 F_{sph}(Qr_i)$ is continuous in the interval of $(R_{min})_i$ and $(R_{max})_i$, where $(R_{min})_i$ and $(R_{max})_i$ define the continuous pore radius corresponding to the discrete pore radius value r_i, let t be the number of subintervals between $(R_{min})_i$ and $(R_{max})_i$. The subinterval width is calculated as $\Delta r_i =$

$[(R_{max})_i - (R_{min})_i]/t$. $\gamma(Q, r_i)$ can then be approximately estimated as the sum of the area of all the subintervals under the $V_r^2 F_{sph}(Q, r_i)$ curve. Mathematically, it is written as

$$\begin{aligned}\gamma(Q, r_i) &\approx \sum_{j=0}^{t-1} V^2_{R_{min_i} + \left(j+\frac{1}{2}\right)\Delta r_i} F_{sph}\left[Q\left(R_{min_i} + \left(j + \frac{1}{2}\right)\Delta r_i\right)\right]\Delta r_i \\ &= \sum_{j=0}^{t-1} Z\left[Q; \left(R_{min_i} + \left(j + \frac{1}{2}\right)\Delta r_i\right)\right] \\ &= \sum_{j=0}^{t-1} Z\left[Q; \left(R_{max_i} - \left(t - j - \frac{1}{2}\right)\Delta r_i\right)\right],\end{aligned} \quad (1)$$

where $R_{min_i} + \left(j + \frac{1}{2}\right)\Delta r_i = R_{max_i} - \left(t - j - \frac{1}{2}\right)\Delta r_i$ is the mid-point of each subinterval j.

This process begins by creating an empty 2D matrix, called matrix α, with the number of rows and columns corresponding to the number of Q and r_i values ($m \times n$, with m representing the number of Q values from the input SAS data and n corresponding to the number of r_i values obtained in the previous step), similar to that of Figure S7. Next, for each entry in the α matrix, a column of t values is generated, where t denotes the number of subintervals for the numerical calculation of $\gamma(Q, r_i)$. This results in a 3D matrix, termed matrix β, as illustrated in Figure S6. Each cell in β is subsequently populated with the corresponding information necessary for the calculation of Z in equation (1): Q, $(R_{min})_i$, $(R_{max})_i$, j, and t (Figure S8 and Figure S9).

As the calculations of Z between the cells of β are identical, they can be performed simultaneously using the aforementioned NumPy vectorised operation, saving significant amounts of calculation time. Once the Z value for each cell in β is determined, a summation along direction **1** of Figure S6 is performed, also using vectorised operations, resulting in the empty matrix α now containing values of $\gamma(Q, r_i)$ corresponding to each pair of $[Q, r_i]$.

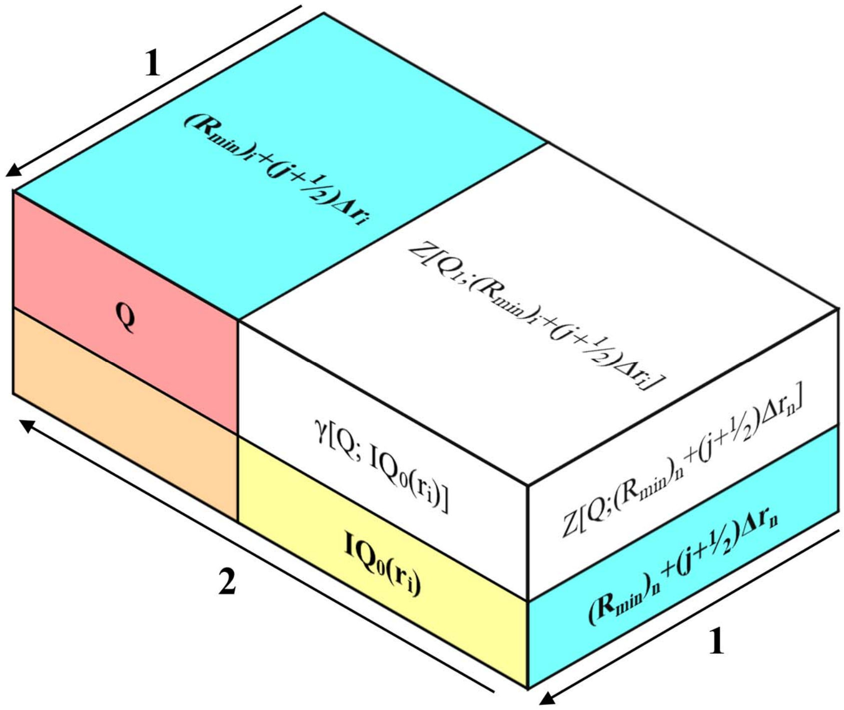

Figure S6 Demonstration of 3D matrix β used for the calculation of integral γ for each pair of Q and $IQ_0(r_i)$. Expanded views of the left, top, and right panels are represented in Figure S7, Figure S8, and Figure S9, respectively. Blue colour indicates the subintervals divided between each pair of $(R_{min})_i$ and $(R_{max})_i$, indexed by the running j values, with each pair of $(R_{min})_i$ and $(R_{max})_i$ corresponding to a value of IQ_{0i}; yellow colour indicating the different values of $IQ_0(r_i)$ and; red colour indicating the different Q values. Arrows labelled **1** indicate the direction in which the integral γ is calculated for each pair of Q and IQ_{0i} following Equation (1); arrow labelled **2** denotes the direction of the intensity calculation for each Q value, estimated using equation (4).

**Q_1**	$\gamma[Q_1, IQ_0(r_1)]$	$\gamma[Q_1, IQ_0(r_2)]$	…	$\gamma[Q_1, IQ_0(r_{n-1})]$	$\gamma[Q_1, IQ_0(r_n)]$
**Q_2**	$\gamma[Q_2, IQ_0(r_1)]$	$\gamma[Q_2, IQ_0(r_2)]$	…	$\gamma[Q_2, IQ_0(r_{n-1})]$	$\gamma[Q_2, IQ_0(r_n)]$
**…**	…	…	…	…	…
**Q_{m-1}**	$\gamma[Q_{m-1}, IQ_0(r_1)]$	$\gamma[Q_{m-1}, IQ_0(r_2)]$	…	$\gamma[Q_{m-1}, IQ_0(r_{n-1})]$	$\gamma[Q_{m-1}, IQ_0(r_n)]$
**Q_m**	$\gamma[Q_m, IQ_0(r_1)]$	$\gamma[Q_m, IQ_0(r_2)]$	…	$\gamma[Q_m, IQ_0(r_{n-1})]$	$\gamma[Q_m, IQ_0(r_n)]$
	**$IQ_0(r_1)$**	**$IQ_0(r_2)$**	**…**	**$IQ_0(r_{n-1})$**	**$IQ_0(r_n)$**

Figure S7 Expanded view of the left panel of Figure S6 (matrix α). Each $IQ_0(r_i)$ value corresponds to a r_i value determined in the Section 4.1; different Q values are obtained from the input SANS data. Each $\gamma(Q, r_i)$ value can be used to calculate the corresponding $\omega(Q, r_i)$ following equation (4), where the summation of all the individual $\omega(Q, r_i)$ in horizontal direction (direction **2** of Figure S6) results in the calculated $I(Q)$ curve, to be fitted to the input SAS data.

**$(R_{max})_i-\frac{1}{2}(\Delta r)_i$**	$Z[Q_1;(R_{max})_1-\frac{1}{2}(\Delta r)_1]$	$Z[Q_1;(R_{max})_2-\frac{1}{2}(\Delta r)_2]$	…	$Z[Q_1;(R_{max})_{n-1}-\frac{1}{2}(\Delta r)_{n-1}]$	$Z[Q_1;(R_{max})_n-\frac{1}{2}(\Delta r)_n]$
**$(R_{max})_i-\frac{3}{2}(\Delta r)_i$**	$Z[Q_1;(R_{max})_1-\frac{3}{2}(\Delta r)_1]$	$Z[Q_1;(R_{max})_2-\frac{3}{2}(\Delta r)_2]$		$Z[Q_1;(R_{max})_{n-1}-\frac{3}{2}(\Delta r)_{n-1}]$	$Z[Q_1;(R_{max})_n-\frac{3}{2}(\Delta r)_n]$
**…**	…	…	…	…	…
**$(R_{min})_i+\frac{3}{2}(\Delta r)_i$**	$Z[Q_1;(R_{min})_1+\frac{3}{2}(\Delta r)_1]$	$Z[Q_1;(R_{min})_2+\frac{3}{2}(\Delta r)_2]$		$Z[Q_1;(R_{min})_{n-1}+\frac{3}{2}(\Delta r)_{n-1}]$	$Z[Q_1;(R_{min})_n+\frac{3}{2}(\Delta r)_n]$
**$(R_{min})_i+\frac{1}{2}(\Delta r)_i$**	$Z[Q_1;(R_{min})_1+\frac{1}{2}(\Delta r)_1]$	$Z[Q_1;(R_{min})_2+\frac{1}{2}(\Delta r)_2]$	…	$Z[Q_1;(R_{min})_{n-1}+\frac{1}{2}(\Delta r)_{n-1}]$	$Z[Q_1;(R_{min})_n+\frac{1}{2}(\Delta r)_n]$

Figure S8 Expanded view of the top panel of Figure S6. Summation of all the Z values in vertical direction results in $\gamma(Q_1, r_i)$, corresponding to the top row of Figure S7.

$Z[Q_1;(R_{min})_n+\frac{1}{2}(\Delta r)_n]$	$Z[Q_1;(R_{min})_n+\frac{3}{2}(\Delta r)_n]$	…	$Z[Q_1;(R_{max})_n-\frac{3}{2}(\Delta r)_n]$	$Z[Q_1;(R_{max})_n-\frac{1}{2}(\Delta r)_n]$
$Z[Q_2;(R_{min})_n+\frac{1}{2}(\Delta r)_n]$	$Z[Q_2;(R_{min})_n+\frac{3}{2}(\Delta r)_n]$	…	$Z[Q_2;(R_{max})_n-\frac{3}{2}(\Delta r)_n]$	$Z[Q_2;(R_{max})_n-\frac{1}{2}(\Delta r)_n]$
…	…	…	…	…
$Z[Q_{m-1};(R_{min})_n+\frac{1}{2}(\Delta r)_n]$	$Z[Q_{m-1};(R_{min})_n+\frac{3}{2}(\Delta r)_n]$	…	$Z[Q_{m-1};(R_{max})_n-\frac{3}{2}(\Delta r)_n]$	$Z[Q_{m-1};(R_{max})_n-\frac{1}{2}(\Delta r)_n]$
$Z[Q_m;(R_{min})_n+\frac{1}{2}(\Delta r)_n]$	$Z[Q_m;(R_{min})_n+\frac{3}{2}(\Delta r)_n]$	…	$Z[Q_m;(R_{max})_n-\frac{3}{2}(\Delta r)_n]$	$Z[Q_m;(R_{max})_n-\frac{1}{2}(\Delta r)_n]$
$\mathbf{(R_{min})_n+\frac{1}{2}(\Delta r)_n}$	$\mathbf{(R_{min})_n+\frac{3}{2}(\Delta r)_n}$	**…**	$\mathbf{(R_{max})_n-\frac{3}{2}(\Delta r)_n}$	$\mathbf{(R_{max})_n-\frac{1}{2}(\Delta r)_n}$

Figure S9 Expanded view of the bottom right panel of Figure S6. Summation of all the Z values in horizontal direction results in $\gamma(Q, r_i)$ where n is the last $IQ_0(r_i)$ entry, corresponding to the right column of Figure S7.

Section S3 User interface explanation and operational instruction.

Section 3.1 Running the software

There are two primary methods to run the software:

(i) **For machines with Python already installed:** Download all the provided scripts and execute the Python script ***run_PRINSAS.py***. This method is cross-platform compatible and independent of the operating system. The scripts were developed and tested using Spyder on Windows and are available at https://github.com/spyder-ide/spyder/releases.

(ii) For Windows machines, use the standalone *.exe* file extracted from the *.zip* package available on the PRINSAS 2.0's GitHub Releases page (https://github.com/spyder-ide/spyder/releases). This version includes all necessary dependencies and runs portably without requiring additional installations. However, as the program is unsigned, it will likely trigger a warning from Windows Defender or other antivirus software. To bypass this warning, follow the following steps during the first run when the antimalware software is triggered (Fig. S10): Click **More info** > Select **Run anyway**. These steps apply to the default Windows 10 antivirus, Microsoft Defender. The process may vary slightly for other antivirus programs or versions of Windows.

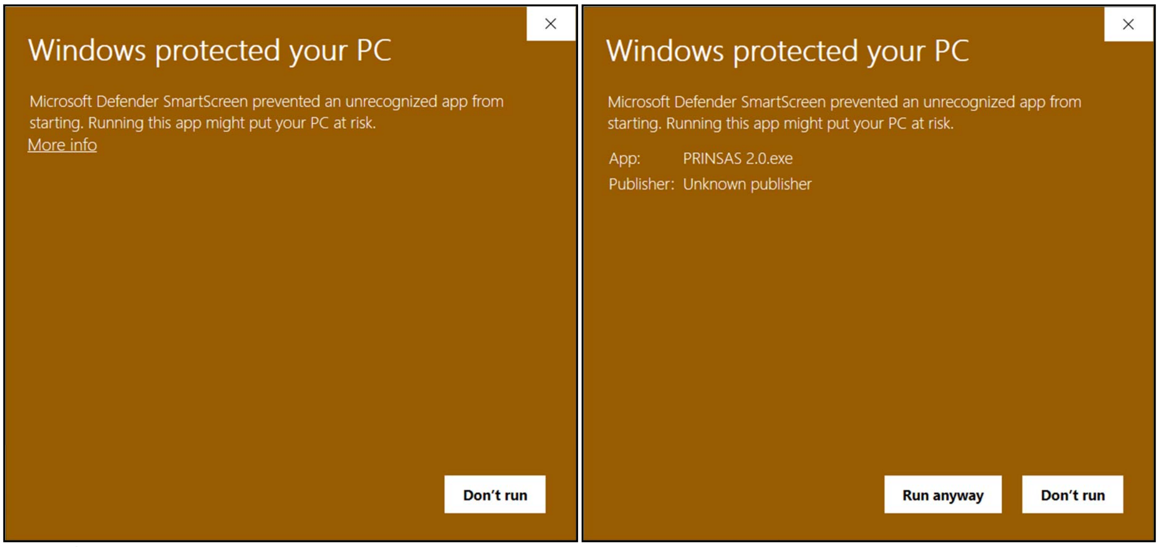

Figure S10 Bypassing Windows Defender in Windows 10 during the first launch of the software.

Section S3.2 Starting user interface

The software interface (Fig. S11) is divided into two primary regions:

- Left: User input/Fitting result area, including:
    - Data Selection Area: Allows users to load data files.
    - ***Fit Parameters*** panel: Provides input fields for fitting parameters.
    - ***PDSP Fit Results*** panel: Displays the results of the fitting process.
- Right: Plotting area, including:
    - ***SAS Data*** panel: Displays the SAS data and facilitates determination of the flat background and Q range.
    - ***SAS Data vs. Fitted Result*** panel: Compares the background-subtracted data with the intensity calculated from the PDSP fit result.
    - ***dV/dr Plot*** and ***f(r) vs. r*** || ***SSA(R) vs. R*** panels: Display the PDSP fit result, showing the volume-weighted pore size distribution, number-weighted pore size distribution and cumulative specific surface area as a function of pore size.

Initially, all buttons are disabled except the ***Choose File*** button on the top left.

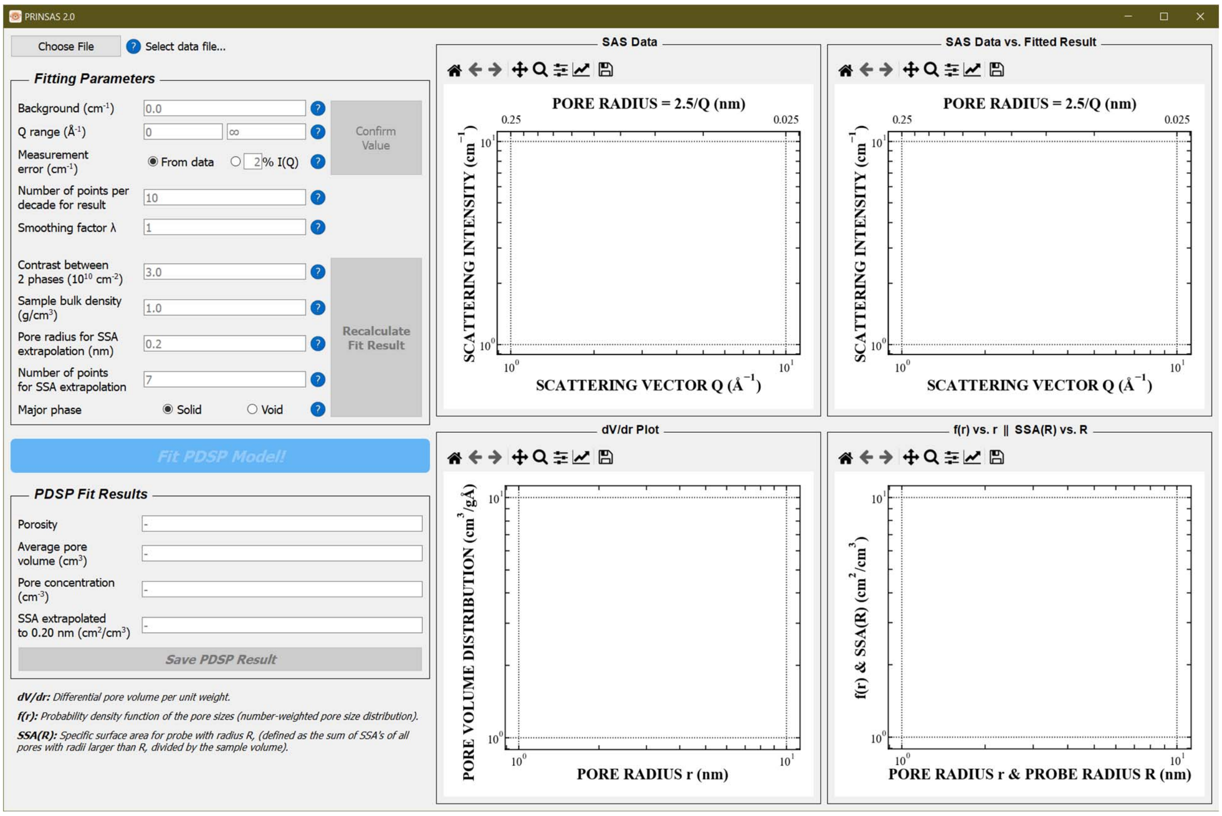

Figure S11 Starting interface layout of the software.

Section S3.3 Selecting data files

To load scattering data for PDSP fitting:

(i) Click the ***Choose File*** button. A pop-up window will appear to allow selection of the desired data file (Fig. S12).

(ii) Once loaded (Fig. S13):

- The file name is displayed next to the ***Choose File*** button.
- The SAS data, including experimental error as error bars, is plotted on the ***SAS Data*** panel. For data files without the optional third column, the error value defaults to 0.
- Background-subtracted SAS data (with the default subtracted background value of 0 cm^{-1}) is also plotted on the ***SAS Data*** and ***SAS Data vs. Fitted Result*** panels.
- The ***Confirm Background*** and ***Fit PDSP Model!*** buttons are enabled.

ASCII data files are read using the function ***read_SANS_data()***, supporting a range of delimiters as well as non-uniform data tables, headers, and footers. The first two columns of data are assigned to Q and $I(Q)$, with the optional third column assigned to $dI(Q)$. However, each file can only use a single delimiter, and decimal numbers in the 'comma' format are not supported (*e.g.*, ***1.234*** is supported, but ***1,234*** is not).

To process data, ***read_SANS_data()*** scans the entire file to identify the most common delimiter. It then splits each row using this delimiter and attempts to convert the elements into numbers, removing any entries that are negative or not a number. Next, it counts the resulting elements of each row and determines the most frequent column count to structure a table. Finally, data rows with insufficient values are discarded.

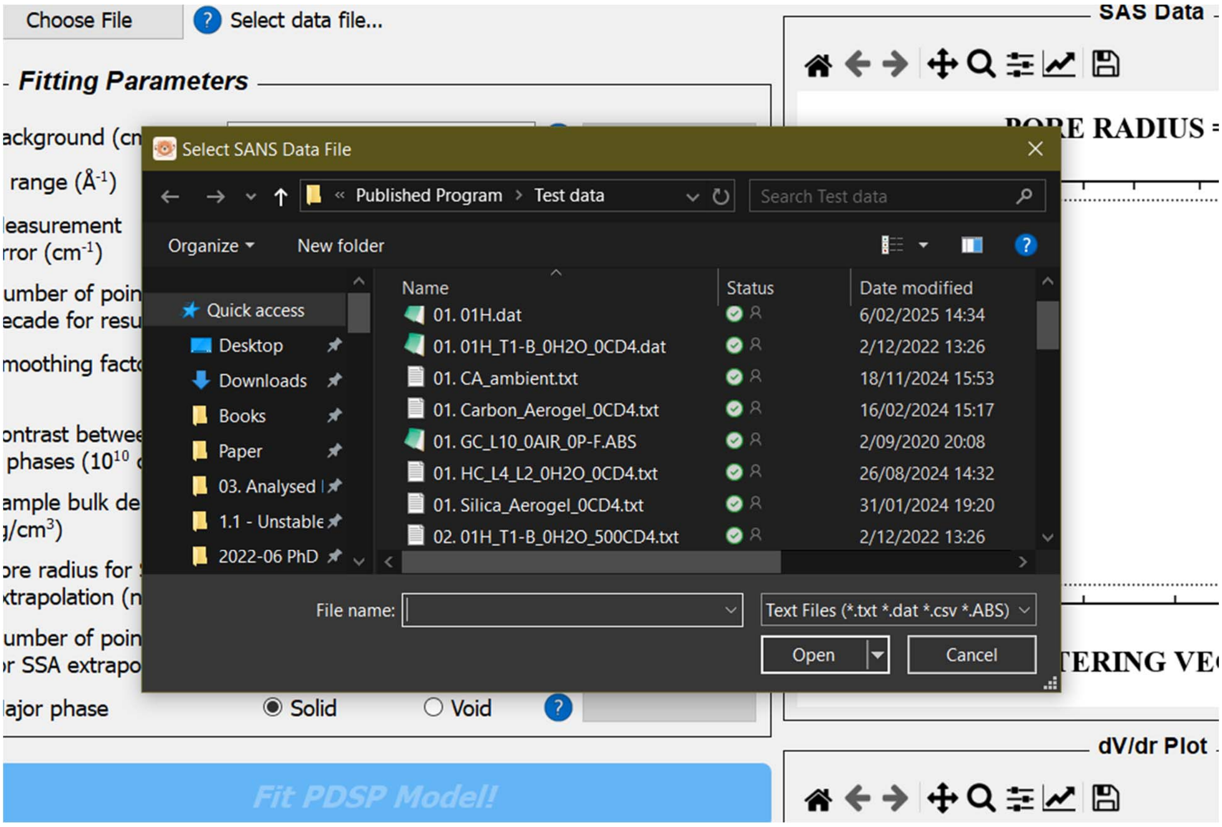

Figure S12 Pop-up window for selecting a data file.

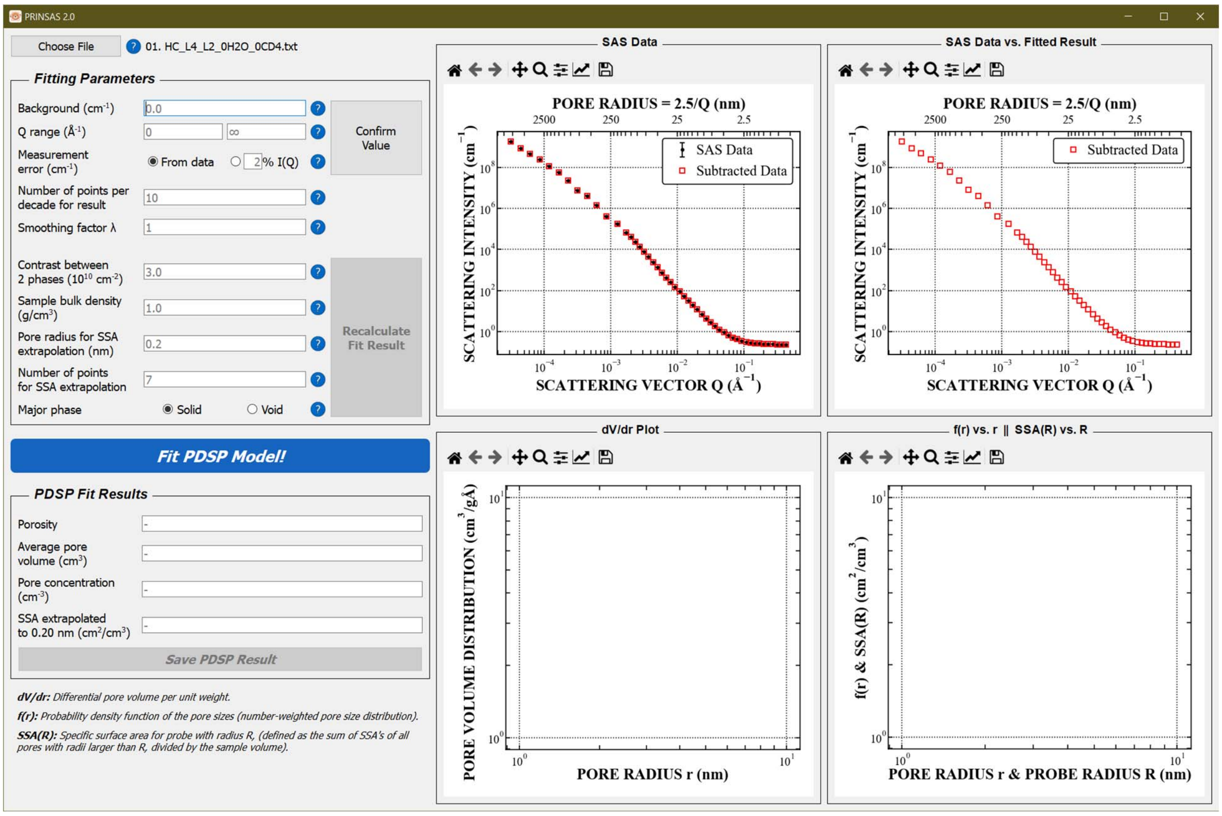

Figure S13 Program interface after loading a scattering data file.

Section S3.4 Configuring fitting parameters and running the fitting procedure

The panel ***Fitting Parameters*** provides the necessary input fields for configuring the PDSP fitting process and calculating the resulting structural properties derived from the fit:

- ***Background (cm^{-1})***: is the flat background value to be subtracted from the original SAS profile prior to the analysis. The background value is determined such that the scattering profile forms a straight, continuous line in the large-Q region after subtraction *(Radlinski et al., 2004b)*. To find the appropriate background value, hover the cursor over the desired level on the ***SAS Data*** plot; live cursor coordinates will be displayed in the top right of the plot (as shown in Fig. S13). The background scattering intensity (y-coordinate) can then be entered into the text box next to the ***Background (cm^{-1})*** label, followed by pressing the ***Confirm Background*** button. This updates the ***SAS Data*** and ***SAS Data vs. Fitted Result*** panels with the newly background-subtracted SAS data (Fig. S14).

- ***Q range (Å^{-1})**:* is the minimum and maximum Q values to be used for the PDSP analysis. The Q range is chosen to exclude SAS data affected by multiple scattering (at low-Q) and artifacts caused by background subtraction (at high-Q). The desired values can be determined using the x-coordinates of the cursor when hovering over the desired position on the ***SAS Data*** plot. Once entered, the selected Q range can be visualised in the ***SAS Data*** and ***SAS Data vs. Fitted Result*** panels by pressing the ***Confirm Background*** button.
- ***Measurement error (cm^{-1})**:* Can be chosen to be either the $dI(Q)$ values acquired from the data file or as a percentage of the scattering intensity $I(Q)$. If no value of $dI(Q)$ is acquired from the input scattering data, percentage error is forced to be used instead.
- ***Number of points per decade for result***: refers to the number of r_i values between each decade of r, where r = $2.5/Q$, in the fit results. A higher number of r_i values improves the fit to the original intensity profile. However, using too many r_i values may lead to overfitting and increase the time required to achieve a complete fit.
- ***Smoothing factor λ**:* Balance the weighting between precisely fitting the input scattering intensity or the continuity of the fit result, with higher λ values leading to smoother fit results.
- ***Contrast between 2 phases (10^{10} cm^{-2})***: refers to the contrast ($\rho_1^* - \rho_2^*$) in Equation (1). For a porous system without any filling medium (*i.e.* the filling medium is vacuum or air), the contrast is equal to the scattering length density (SLD) of the solid.
- ***Sample bulk density (g/cm^3)***: required for the differential pore volume distribution calculations—dV/dr—as outlined in Equation (12).
- ***Pore radius for SSA extrapolation (nm)***: is the pore radius to which the SSA value is extrapolated in the $SSA(R)$ vs. R graph, represented by the vertical dashed line on the $SSA(R)$ vs. R plot in Figure 5. The default R value is 0.2 nm, based on the study of Radlinski et al. *(Radlinski et al., 2004b)*.
- ***Number of points for SSA extrapolation***: refers to the number of data points used for the SSA extrapolation to the previously specified pore radius in the $SSA(R)$ vs. R graph, indicated by the solid blue marker on the $SSA(R)$ vs. R plot in Fig. S15.
- ***Major phase*** (Void or Solid): since equation (1) is symmetric for both phases of ϕ and $(1 - \phi)$, the phase that accounts for the majority of the system's volume must be selected to ensure that the correct porosity value is returned.

Once all the inputs have been entered, the ***Fit PDSP Model!*** button will save the provided input parameters and initiate the PDSP fit procedure. Any values left blank will be assigned the default, arbitrarily chosen based on the authors' experience working with various geological samples, which is displayed in light grey when no input is provided. The fitting and calculation process may take up to a few minutes, depending on the Q range and the number of data points in the selected SAS data, as well as the input for ***Number of points per decade for result***. During this time, the program becomes unresponsive.

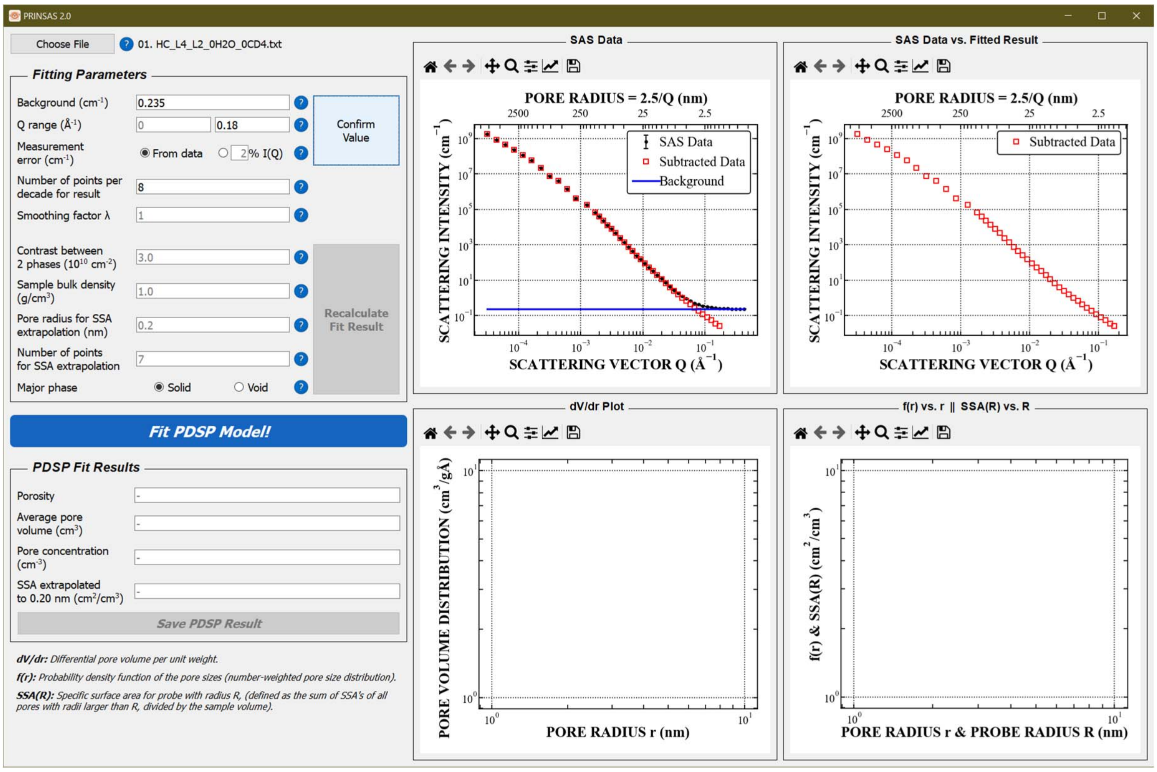

Figure S14 Program interface after background and Q range values have been specified.

Section 3.5 Fit result and recalculating structural properties post-fitting

Once the fitting procedure is completed (Fig. S15), the calculated results are presented in the ***PDSP Fit Result*** panel including:

- Porosity (ϕ)
- Average pore volume ($\bar{V}$)

- Pore concentration/number density ($\phi / \bar{V}$)
- SSA value extrapolated to the previously specified ***Pore radius for SSA extrapolation***.

The software also plots the fitted $I(Q)$ value to the ***SAS Data vs. Fitted Result*** panel for comparison with the background-subtracted SAS data, and displays dV/dr, $f(r)$, and $SSA(R)$ results in their respective plot panels.

Note that these values are highly dependent on the absolute scattering intensity, which is not always reliable, especially for powdered samples.

After the fit, the ***Recalculate Fit Result*** button is enabled, allowing for the recalculation of the structural properties within the ***PDSP fit result*** without repeating the entire fitting process. This recalculation can be applied when the following inputs are changed: ***Contrast between 2 phases***, ***Sample bulk density***, ***Pore radius for SSA extrapolation***, ***Number of points for SSA extrapolation***, and ***Major phase***. After adjusting these values, clicking the ***Recalculate Fit Result*** button updates the results and plots accordingly (Fig. S16).

Once the fitting procedure is complete, the ***Fit PDSP Model!*** button is disabled. It will be re-enabled only if (i) a new background, Q range, or measurement error are selected, or (ii) a different number of r_i per decade or λ are input.

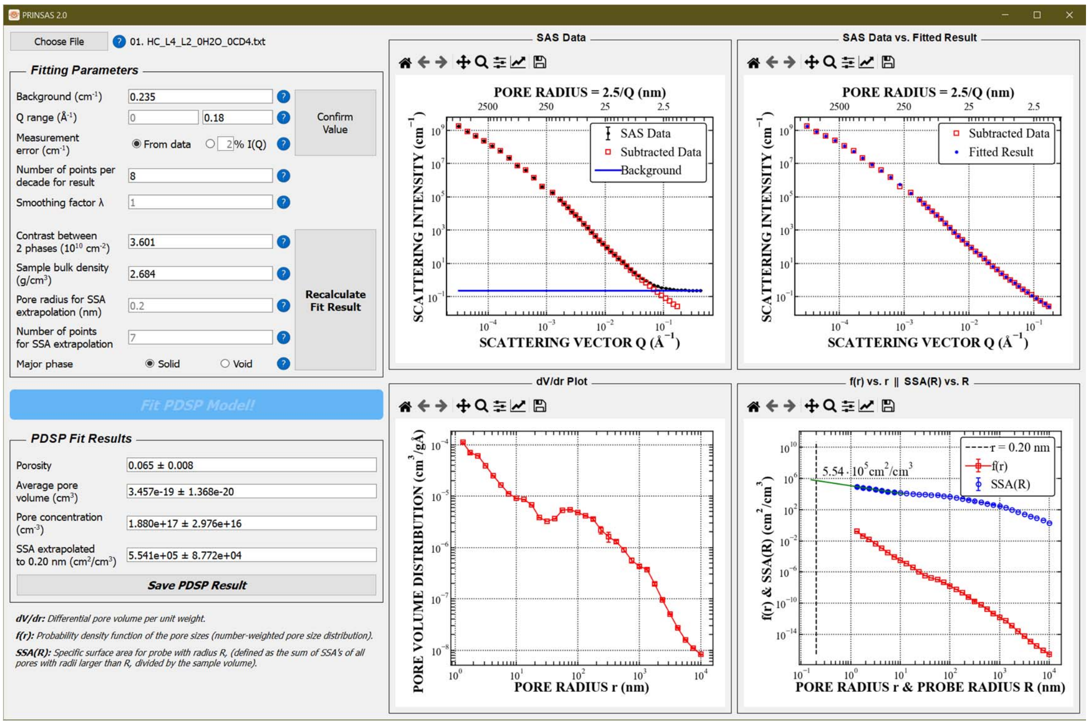

Figure S15 Program interface after the PDSP fitting procedure is completed.

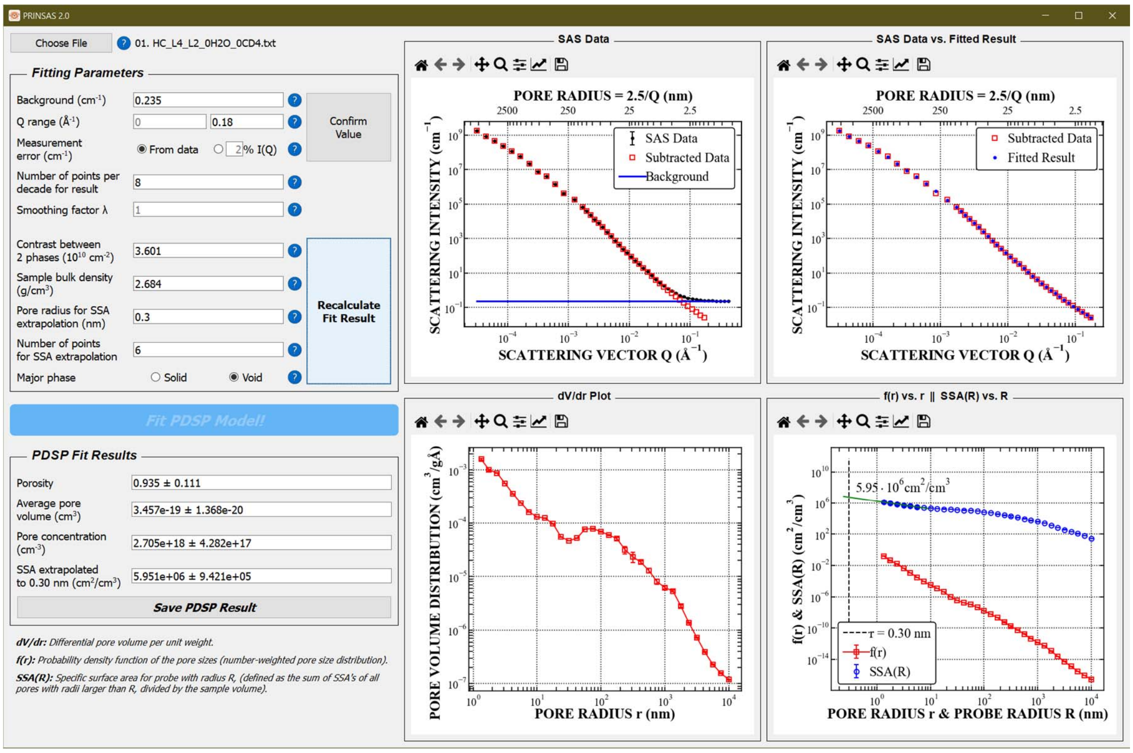

Figure S16 Recalculation of results after entering new inputs (Pore radius for SSA extrapolation, Number of points for SSA extrapolation, and Major phase) without refitting.

Section 3.6 Saving the result

The fit results can be saved to ASCII files generated by the program. These files include all the fitting parameters in addition to the fit result (Fig. S18). To save the result, press the ***Save PDSP Result*** button (enabled after the fitting procedure is completed). A pop-up will appear, allowing the user to choose where to save the file (Fig. S17).

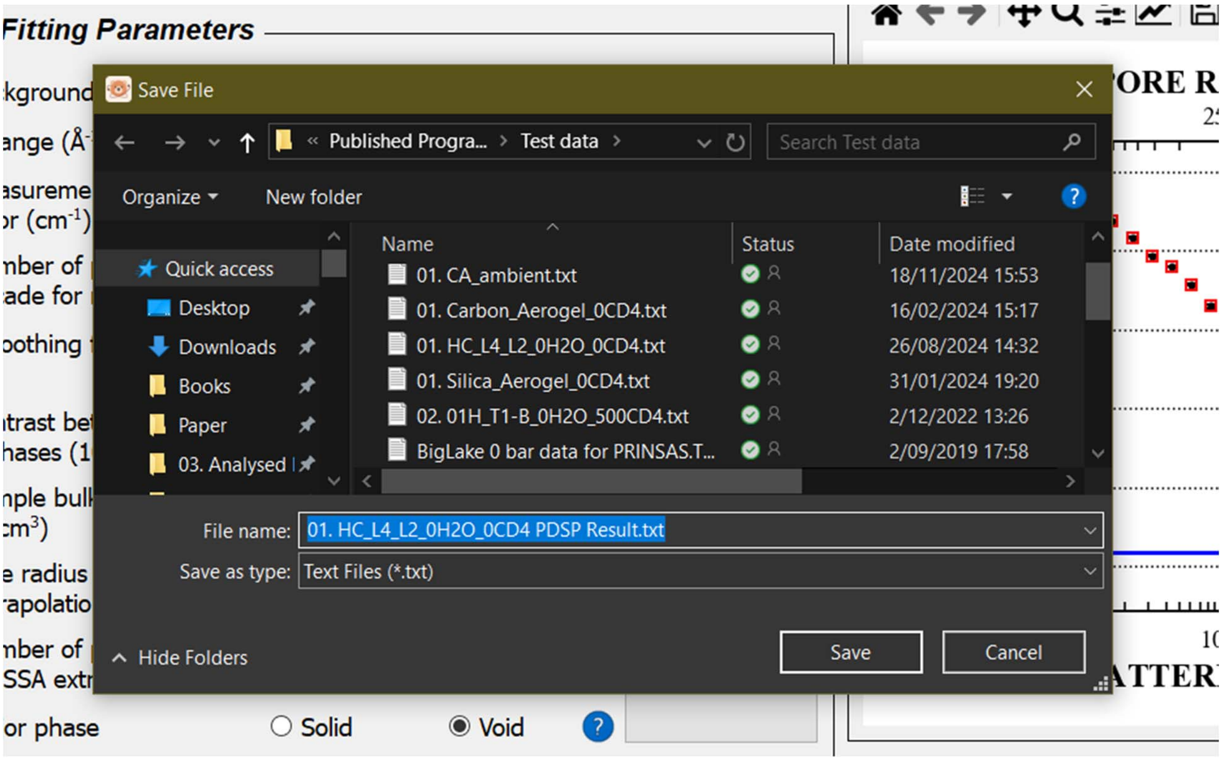

Figure S17 Pop-up window for saving results.

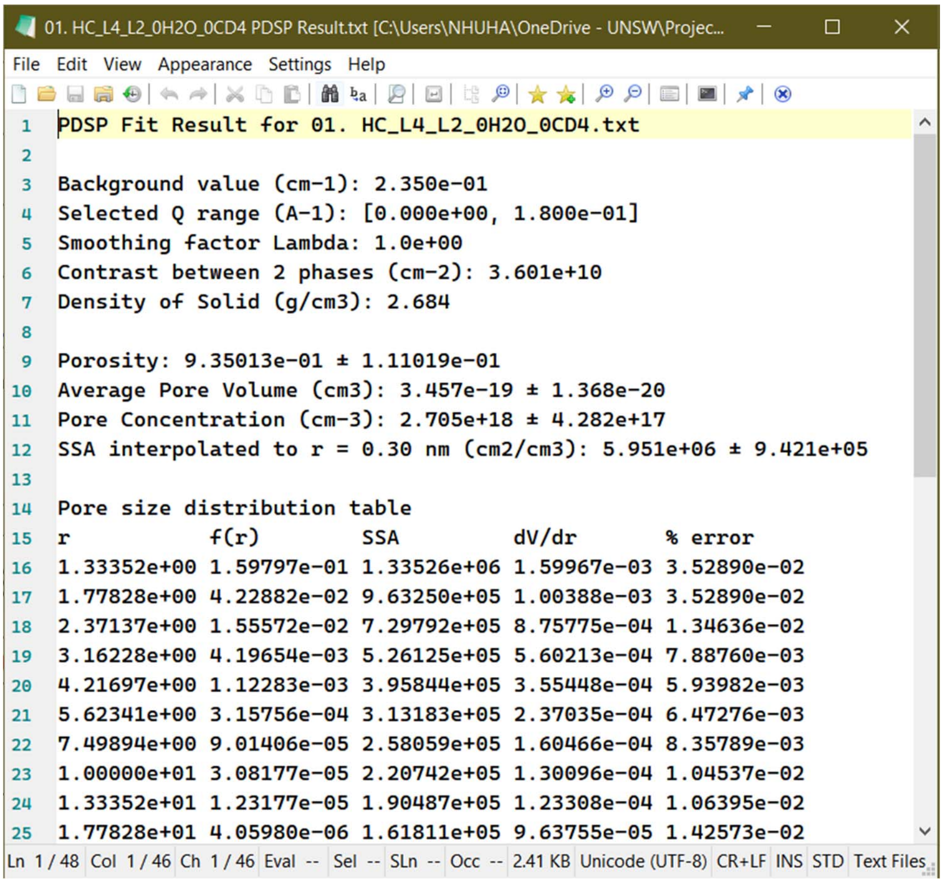


```
PDSP Fit Result for 01. HC_L4_L2_0H2O_0CD4.txt

Background value (cm-1): 2.350e-01
Selected Q range (A-1): [0.000e+00, 1.800e-01]
Smoothing factor Lambda: 1.0e+00
Contrast between 2 phases (cm-2): 3.601e+10
Density of Solid (g/cm3): 2.684

Porosity: 9.35013e-01 ± 1.11019e-01
Average Pore Volume (cm3): 3.457e-19 ± 1.368e-20
Pore Concentration (cm-3): 2.705e+18 ± 4.282e+17
SSA interpolated to r = 0.30 nm (cm2/cm3): 5.951e+06 ± 9.421e+05

Pore size distribution table
r           f(r)        SSA         dV/dr       % error
1.33352e+00 1.59797e-01 1.33526e+06 1.59967e-03 3.52890e-02
1.77828e+00 4.22882e-02 9.63250e+05 1.00388e-03 3.52890e-02
2.37137e+00 1.55572e-02 7.29792e+05 8.75775e-04 1.34636e-02
3.16228e+00 4.19654e-03 5.26125e+05 5.60213e-04 7.88760e-03
4.21697e+00 1.12283e-03 3.95844e+05 3.55448e-04 5.93982e-03
5.62341e+00 3.15756e-04 3.13183e+05 2.37035e-04 6.47276e-03
7.49894e+00 9.01406e-05 2.58059e+05 1.60466e-04 8.35789e-03
1.00000e+01 3.08177e-05 2.20742e+05 1.30096e-04 1.04537e-02
1.33352e+01 1.23177e-05 1.90487e+05 1.23308e-04 1.06395e-02
1.77828e+01 4.05980e-06 1.61811e+05 9.63755e-05 1.42573e-02
```

Figure S18 Example of a saved result file.

Section 3.7 Plotting window functionality

Each plotting window includes functionalities to facilitate the inspection and visualisation of the SAS data and PDSP fit results. In addition to displaying live cursor coordinates, as mentioned previously, the toolbar located to the left of the coordinates provides the following features (arranged from left to right):

- ***Home:*** Resets the plot to its original state.
- ***Back/Forward:*** Undoes or redoes adjustments made to the plot.
- ***Pan:*** Enables panning functionality.
- ***Zoom:*** Allows zooming functionality.
- ***Configure subplots:*** Adjusts the spacing and border of the plotting area relative to the window frame.

- ***Edit axis/curve:*** Provides options for customising plot elements, such as the plot title, axis limits, line and marker shape or colour, and other properties of the data series. However, this function has two limitations:
    - The inability to customise error plots, as this feature is embedded in the plotting and rendering engine Matplotlib (referenced at https://github.com/matplotlib/matplotlib/issues/18631).
    - For SAS plots, the values on the top and bottom axes (scattering vector Q and corresponding pore radius ≈ 2.5/Q) are not automatically linked. They must be adjusted independently when changing the range of horizontal axis. To ensure the r values on the top axis match the Q values on the bottom axis, update the top axis limits using the following formulas: $(x_{min})_{top} = 0.1 / (x_{max})_{bottom}$; $(x_{max})_{top} = 0.1 / (x_{min})_{bottom}$. This issue occurs only when modifying the axes using the ***Edit axis/curve*** function. It does not affect the zoom or pan functionality.

- ***Save:*** Exports the plot in various image formats (*.png*, *.jpeg*, *.svg*, *etc.*)
